# Synthesis and Structure-Activity Relationship Analysis of 5-HT_7_ Receptor Antagonists: Piperazin-1-yl Substituted Unfused Heterobiaryls

**DOI:** 10.3390/molecules21040433

**Published:** 2016-03-31

**Authors:** Lucjan Strekowski, Jarosław Sączewski, Elizabeth A. Raux, Nilmi T. Fernando, Jeff Klenc, Shirish Paranjpe, Aldona Raszkiewicz, Ava L. Blake, Adam J. Ehalt, Samuel Barnes, Timothy C. Baranowski, Shannon M. Sullivan, Grzegorz Satała, Andrzej J. Bojarski

**Affiliations:** 1 Department of Chemistry, Georgia State University, Atlanta, Georgia, GA 30302, USA; earaux@gmail.com (E.A.R.); fernando.nilmi@gmail.com (N.T.F.); jklenc@merchantgould.com (J.K.); shirishp007@gmail.com (S.P.); aldona.paluchowska@urpl.gov.pl (A.R.); ablake@bakerlaw.com (A.L.B.); adamehalt@gmail.com (A.J.E.); sambarnes2@gmail.com (S.B.); tcb@bellsouth.net (T.C.B.); ssullivan@yahoo.com (S.M.S.); 2 Department of Chemical Technology of Drugs, Medical University of Gdańsk, Aleja Generała Józefa Hallera 107, Gdańsk 80-416, Poland; 3 Department of Medicinal Chemistry, Institute of Pharmacology, Polish Academy of Sciences, Smętna 12, Kraków 31-343, Poland; satala@if-pan.krakow.pl (G.S.); bojarski@if-pan.krakow.pl (A.J.B.)

**Keywords:** 5-HT_7_ receptor ligands, 5-HT, serotonin, synthesis, structure-affinity relationships (SAR), *N*-methylpiperazine, 3-furyl

## Abstract

A series of piperazin-1-yl substituted unfused heterobiaryls was synthesized as ligands of the 5-HT_7_ receptors. The goal of this project was to elucidate the structural features that affect the 5-HT_7_ binding affinity of this class of compounds represented by the model ligand 4-(3-furyl)-2-(4-methylpiperazin-1-yl)pyrimidine (**2**). The SAR studies included systematical structural changes of the pyrimidine core moiety in **2** to quinazoline, pyridine and benzene, changes of the 3-furyl group to other heteroaryl substituents, the presence of various analogs of the 4-methylpiperazin-1-yl group, as well as additional substitutions at positions 5 and 6 of the pyrimidine. Substitution of position 6 of the pyrimidine in the model ligand with an alkyl group results in a substantial increase of the binding affinity (note a change in position numbers due to the nomenclature rules). It was also demonstrated that 4-(3-furyl) moiety is crucial for the 5-HT_7_ binding affinity of the substituted pyrimidines, although, the pyrimidine core can be replaced with a pyridine ring without a dramatic loss of the binding affinity. The selected ethylpyrimidine (**12**) and butylpyrimidine (**13**) analogs of high 5-HT_7_ binding affinity showed antagonistic properties in cAMP functional test and varied selectivity profile—compound **12** can be regarded as a dual 5-HT_7_/5-HT_2A_R ligand, and **13** as a multi-receptor (5-HT_7_, 5-HT_2A_, 5-HT_6_ and D_2_) agent.

## 1. Introduction

For more than 60 years, the studies on the role of serotonin (5-HT) within the central nervous system (CNS) have constantly provided evidence of the involvement of 5-HT receptors in the action of various psychiatric drugs. It is now evident that deregulation of serotonergic signaling is associated with pathogenesis of severe neuropsychological disorders like depression [1,2] Alzheimer’s disease [3,4] or schizophrenia [5], and not surprisingly the 5-HT signaling system is one of the major focuses of various CNS drug development strategies [6].

Among the plethora of functions influenced by fourteen 5-HT receptor subtypes, activation of 5-HT_7_ receptors (5-HT_7_R) in the CNS regulates circadian rhythms (facilitates diurnal phase shift and REM sleep) [7,8], thermoregulation (induces hypothermia) [9,10] and cognitive processes by facilitation of long and short-term memory consolidation [11,12,13]. Moreover, the presence of 5-HT_7_ receptors in key cerebral areas as well as high affinity of numerous antidepressants and antipsychotics for the 5-HT_7_ receptors, indicate their potential role in depression and schizophrenia [14,15,16,17]. The results of pre-clinical studies have shown that both genetic inactivation and pharmacological blockade of the 5-HT_7_ receptors leads to antidepressant-like effects in animal models of depression [14,16]. Similarly, antagonism at 5-HT_7_ receptors is one of the key pharmacological features of vortioxetine, an atypical antidepressant that was approved for medication in 2013 by the U.S. FDA [18]. Therefore, the new individual therapies targeting the blockade of 5-HT_7_ receptors in combination with using antidepressants are currently under investigation [19].

In relation to schizophrenic disorders, the involvement of 5-HT_7_ receptors in the pro-social action of amisulpride has been recently demonstrated, and 5-HT_7_ receptor antagonism was postulated as a possible mechanism for the treatment of social withdrawal—one of the core negative symptoms of schizophrenia [20]. Other preclinical findings, implicating the role of 5-HT_7_ receptors in learning and memory, suggest that blockade of this receptor may be beneficial against cognitive impairments in schizophrenia [21].

Given the role of 5-HT_7_ receptors in the mechanism of action of antidepressants and antipsychotics, various groups of ligands have been developed but attributed mainly with linear arrangement of basic pharmacophore features that include long chain arylpiperazine structure [22,23]. Looking for new leads with different pattern of potential ligand-5-HT_7_ receptor interactions, we screened our in-house compound library and high 5-HT_7_R affinity was found for some 4-mono-, or 4,6-di-substutited 2-(4-methylpiperazino)pyrimidine derivatives, developed earlier as 5-HT_2A_ antagonists [24]. Here we present synthesis and extensive structure-5-HT_7_ receptor affinity study on piperazin-1-yl substituted unfused heterobiaryls and related compounds.

## 2. Results

### 2.1. Chemistry

**Scheme 1:**
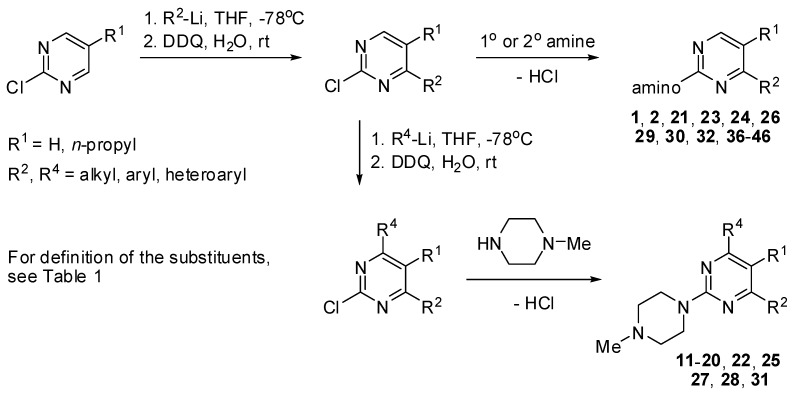
Synthesis of substituted pyrimidines **1**, **2**, **11**–**28**, **36**–**46** (see Table 1).

**Scheme 2:**
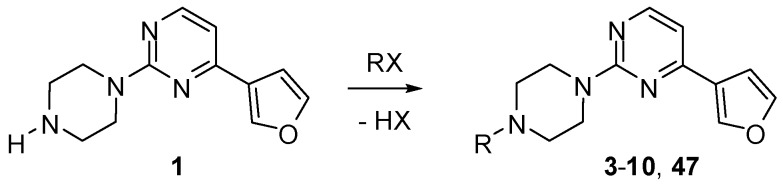
Synthesis of substituted pyrimidines **3**–**10** and **47** (see Table 1).

The commercially available substituted pyrimidines, quinazolines, pyridines and benzenes were used as starting materials in the synthesis of a novel library of 5-HT_7_ receptor ligands. Substituted pyrimidines are the most extensive class of compounds used in this study. The general synthetic approach developed in our laboratories includes the addition reaction of an aryl(heteroaryl)lithium reagent to generate a substituted dihydropyrimidine or dihydroquinazoline intermediate product, which, without isolation, undergoes aromatization upon treatment with 2,3-dichloro-5,6-dicyano-1,4-benzoquinone (DDQ) (Scheme 1 and Scheme 2) [24,25].

**Scheme 3:**
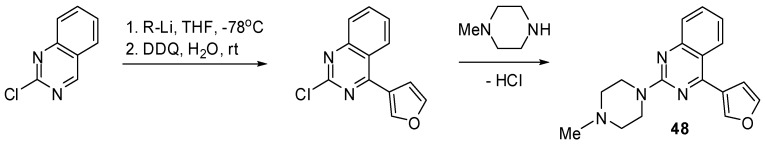
Synthesis of quinazoline **48**.

**Scheme 4:**
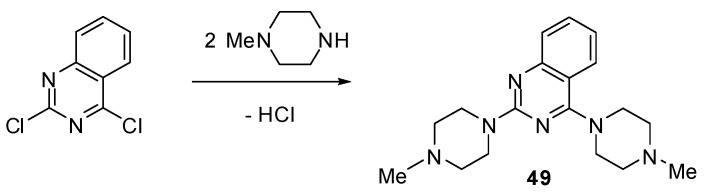
Synthesis of quinazoline **49**.

**Scheme 5:**
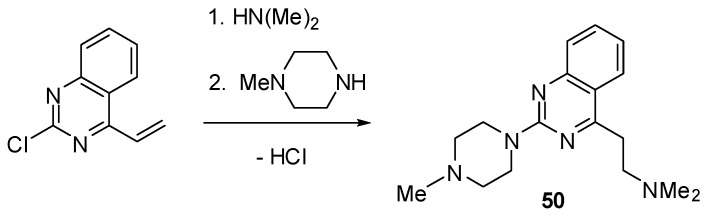
Synthesis of quinazoline **50**.

**Scheme 6:**
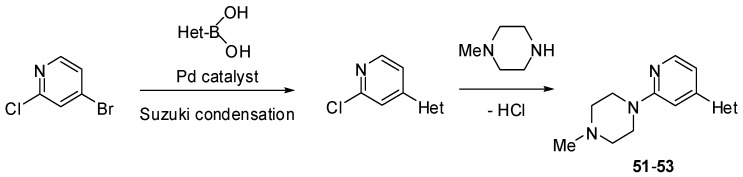
Synthesis of substituted pyridines **51**–**53** (see Table 4).

**Scheme 7:**
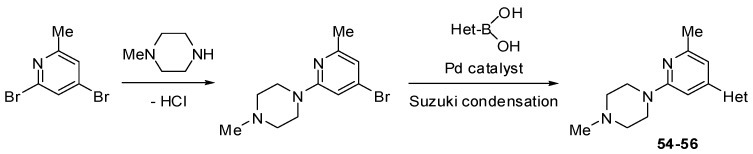
Synthesis of substituted pyridines **54**–**56** (see Table 4).

**Scheme 8:**
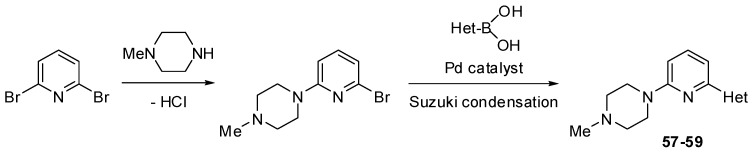
Synthesis of substituted pyridines **57**–**59** (see Table 4).

Many final products were obtained by nucleophilic displacement reaction of a halogen activated by an appropriately positioned ring nitrogen atom in pyrimidines, quinazolines and pyridines (Scheme 1 and Scheme 3, Scheme 4, Scheme 5, Scheme 6, Scheme 7 and Scheme 8). By using this methodology, 4-substituted 2-chloropyrimidine or 2-chloroquinazoline substrates were easily functionalized with amines to give the final products **1**, **2**, **21**, **23**, **24**, **26**, **29**, **30**, **32**, **36**–**46**, and **48**. With 2,4-dichloropyrimidines, such nucleophilic displacement is regioselective, giving rise to 4-amino-2-chloropyrimidines, often with yields exceeding 90% [26]. In this work, the regioselective displacement reaction of 2,4-dichloro-6-(3-furyl)pyrimidine with dimethylamine at ambient temperature yielded a 2-chloro-4-dimethylamino intermediate product, the subsequent treatment of which with *N*-methylpiperazine at an elevated temperature furnished the final product 4-dimethylamino-6-(3-furyl)-2-(4-methylpiperazin-1-yl)pyrimidine (**19**). On the other hand, two consecutive introductions of heteroaryl groups at positions 4 and 6 of 2-chloropyrimidine followed by nucleophilic displacement of the 2-chloro atom yielded a variety of substituted pyrimidines **11**–**20**, **22**, **25**, **27**, **28**, **31** (Scheme 1).

The classical alkylation and acylation reactions of **1** provided facile entry to compounds **3**–**10** (Scheme 2). A similar strategy afforded a piperazinium derivative **47** (see Experimental). It was also found that the conjugate addition reaction of dimethylamine with 2-chloro-4-vinylquinazoline [27,28] proceeds cleanly to furnish 2-chloro-4-(2-dimethylamino)ethyl substituted quinazoline, the subsequent treatment of which with *N*-methylpiperazine yielded the desired product **50** (Scheme 5).

The Suzuki mono-coupling reaction of a dihalogeno-substituted pyridine with a heteroarylboronic acid followed by classical nucleophilic displacement of the second halogen were used to synthesize pyridine derivatives **51**–**59** (Scheme 6, Scheme 7 and Scheme 8). The reaction sequence depended on type of substrate used.

**Scheme 9:**
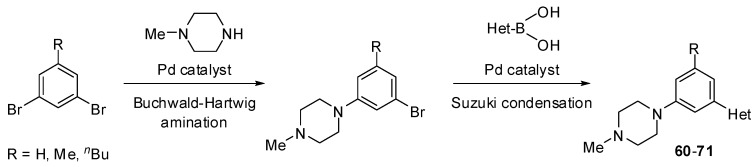
Synthesis of substituted benzenes **60**–**71** (see Table 5).

In case of benzene derivatives, Buchwald-Hartwig amination of halogeno-substituted benzenes with *N*-methylpiperazine in the presence of a palladium catalyst provided piperazine-substituted products (Scheme 9). The Suzuki condensation of these intermediate products with heteroarylboronic acids yielded the desired heteroaryl-substituted benzenes **60**–**71**. It should be noted that the majority of the two-step reactions discussed above were conducted with crude intermediate products. However, the final products were rigorously characterized in all cases studied.

### 2.2. Pharmacology

Novel scaffold endowed with 5-HT_7_R activity was identified by screening of the in-house compound library in a single compound concentration of 1 µM in a competition radioligand binding assay using [^3^H]5-CT and HEK293 cells overexpressing the human 5-HT_7b_ receptors. Compounds showing >60% of inhibition, namely (4-(3-furyl)-2-(4-methylpiperazin-1-yl)pyrimidine (**2**) and its two derivatives **11** and **18** were further tested in full binding experiments, and, as for all the newly synthesized compounds (**1**, **3**–**10**, **12**–**17** and **19**–**71**), 5-HT_7_R affinity (*K*_i_ values ± SD) was determined in at least two independent experiments run in triplicate [31]. The functional properties of two selected compounds **12** and **13** active on 5-HT_7_R were further evaluated using their ability to inhibit cAMP production induced by 5-CT (10 nM) in the same cellular system [32,33]. In addition, compounds **12** and **13** were evaluated for their affinity at three other serotonergic receptors: 5-HT_1A_, 5-HT_2A_, 5-HT_6_ and dopaminergic D_2_ in standard competition binding assays by use of the appropriate radioligand: [^3^H]-8-OH-DPAT (187 Ci/mmol), [^3^H]-Ketanserin (66.9 Ci/mmol), [^3^H]-LSD (85.2 Ci/mmol) and [^3^H]-Raclopride (74.4 Ci/mmol), respectively, and cell line with overexpressed receptor (CHO for 5-HT_2A_ and HEK293 for all the others) [33,34].

## 3. Discussion

The objective of this study was to determine which combinations of structural features affect the 5-HT_7_ binding affinity of unfused heterobiaryls. The intended SAR studies included synthesis of new analogs of the model compound **2** and encompassed the following points: (1) substitution of the amino group within the piperazine ring; (2) introduction of alkyl, amino and bulky substituents into positions 4/6 and/or 5 of the pyrimidine ring; (3) modifications of the heteroaryl group in position 4/6 of the pyrimidine; (4) replacement of the piperazinyl group by other amino substituents; and (5) synthesis of a series of pyridines and benzenes allowing comparison of the influence of the central ring nitrogen atom(s) on the ligand affinity.

First, investigating the influence of substituents at the basic piperazine atom, it was found that unsubstituted (**1**), simple alkyls (**3** and **4**), arylalkyl (**7**), hydroxyalkyl (**8**) or even esters (**9** and **10**) provide a similar level of 5-HT_7_R affinity (*K*_i_ = 1.4–10 nM) as methylated hit compound **2** (*K*_i_ = 7.2 nM). Slight decrease in 5-HT_7_R affinity is observed for benzyl derivative **5** (*K*_i_ = 25 nM) which is probably caused by a negative steric influence of the phenyl group on the key interaction between a protonated nitrogen and aspartic acid (D3.32) residue in the receptor [35]. Moreover, with the benzoyl group (**6**), there is an obvious negative effect on the affinity (*K*_i_ = 1405 nM) that arises from diminished basicity of the piperazine nitrogen atom (Table 1).

In the literature, piperazine has been often a key element of 5-HT_7_R ligands [22], and it is also optimal for heterobiaryl compounds, as its replacement results in decreased affinity (Table 2). Indeed, introduction of homopiperazine leads to ligand with a moderate affinity for 5-HT_7_R (**42**, *K*_i_ = 148 nM), whereas no affinity is observed for piperidine (**43**) and morpholine (**44**) derivatives that lack a basic nitrogen atom. In the group of compounds incorporating sterically hindered amines (**45**–**47**), only the ligand with positively charged quaternary nitrogen atom displayed moderate 5-HT_7_R affinity (**47**, *K*_i_ = 299 nM).

Further modifications of the model compound **2** involved introductions of substituents at position 6 of the pyrimidine ring. Alkyl groups (**11**–**16**) are very well tolerated in terms of 5-HT_7_ receptors affinity (*K*_i_ values in the range of 1.6 to 8.3 nM), while introduction of bulky cyclohexenylethynyl (**17**) and 3-furyl (**18**) groups results only in a minor reduction of the binding affinity with the *K*_i_ values of 18 and 22 nM, respectively. It can be seen, however, that amino groups (**19**, **20**) at position 6 and alkyl substituents at position 5 (**21**, **22**) are highly detrimental for the affinity.

Next, the SAR analysis of the substituents at position 4 of the pyrimidine ring was investigated by modifications and replacement of the 3-furyl group. Compounds **23**, **24** with methyl substituents within the 3-furyl ring display reduced affinities (*K*_i_ values of 1,920 and 462 nM, respectively). Interestingly, introduction of an *n*-hexyl group (**25**) at position 6 of the pyrimidine in compound **24** restores high affinity for 5-HT_7_R (*K*_i_ = 12 nM). A similar trend can be recognized for less active ligands **26** (2-furyl, *K*_i_ = 1,021 nM) and **30** (3-thienyl, *K*_i_ = 83 nM), where addition of a 6-alkyl substituent results in a 2- or 3-fold boost in affinity (ethyl, **27**, *K*_i_ = 380 nM; *n*-butyl, **28**, *K*_i_ = 450 nM and *n*-butyl, **31**, *K*_i_ = 15 nM). As a general rule, 3-furyl (**2**, **24**) and 3-thienyl (**30**) derivatives are 5 to 140-fold more active than their 2-furyl (**26**, **29**) and 2-thienyl (**32**) counterparts. Replacement of the 3-furyl group for *o*-, *m*-, *p*-hydroxyphenyl (**33**–**35**), pyridin-3-yl (**39**), thiazol-2-yl (**40**) or imidazol-1-yl (**41**) group as well as benzo-fused heterocyclic (**36**–**38**) moieties does not furnish ligands with noteworthy affinity for 5-HT_7_R (*K*_i_ > 1000 nM). Thus, it appears that the 3-furyl group is necessary for a high affinity of 5-HT_7_R ligands or, alternatively, a 3-thienyl moiety but with an appropriate enhancing substitutent at position 6 of the pyrimidine ring (Table 1).

Next it was found that quinazoline derivatives (**48**–**50**), which can be treated as 5,6-disubstituted pyrimidine analogs, were inactive (*K*_i_ > 1910 nM). This result is fully consistent with our finding that substitution at position 5 is highly detrimental for 5-HT_7_R affinity (Table 3).

Having established reasonable SAR around pyrimidine central ring, we focused our attention on the role of the pyrimidine core itself, analyzing a series of substituted pyridine- and phenyl-piperazine analogs. The pyridine derivatives (**51**–**59**) were then synthesized to determine whether both nitrogen atoms within the central heterocycle are needed for the ligand to retain affinity for the binding pocket of the receptor (Table 4). The biological data show that both isomeric 4-(3-furyl) (**51**) and 6-(3-furyl) (**57**) pyridine ligands still demonstrate high affinities for the 5-HT_7_R (*K*_i_ = 17 nM and 8 nM, respectively). However, for the 2-furyl derivatives, position 4 (**52** *K*_i_ = 307 nM) is preferred over position 6 (**59** *K*_i_ = 1135 nM) of the pyridine as the attachment site. The pyridine ligands incorporating 3-thienyl (**58** *K*_i_ = 79 nM) and 2-thienyl (**53** *K*_i_ = 396 nM) substituents are nearly equipotent compared to their pyrimidine counterparts (**30**, *K*_i_ = 83 nM and **32**, *K*_i_ = 480 nM). Similarly to pyrimidine ligands, introduction of an alkyl substituent into position 6 of the pyridine ring results in 2- or 3-fold boost in affinity (**54** *K*_i_ = 8 nM; 55 *K*_i_ = 139 nM and **56** *K*_i_ = 117 nM vs **51**, *K*_i_ = 17 nM; **52** *K*_i_ = 307 nM and **53** *K*_i_ = 396 nM, respectively).

After finding that the pyrimidine core can be safely replaced with a pyridine ring, our attention was turned to compounds devoid of any nitrogen atoms within the central moiety of a ligand (Table 5). 3-Furyl (**60**, *K*_i_ = 31 nM) and 3-thienyl (**66**, *K*_i_ = 342 nM) benzene analogs show significant 5-HT_7_R affinities, although they are 2- and 4-fold less active than their pyrimidine and pyridine equivalents. 5-HT_7_R affinities of 2-furyl (**63**, *K*_i_ = 914 nM) and 2-thienyl (**69**, *K*_i_ = 542 nM) derivatives remain at moderate levels. Consequently, introduction of methyl groups into position 5 of 1,3-disubstituted benzene analogs results in 2- to 7-fold increases of the binding properties (**64**, *K*_i_ = 312 nM; **67**, *K*_i_ = 47 nM and **70**, *K*_i_ = 297 nM), except for the 3-furyl ligand (**61**, *K*_i_ = 37 nM) which stays equipotent to its unsubstituted counterpart. Surprisingly, the *n*-butyl substituted benzene derivatives are lower in 5-HT_7_R affinity (**62**, *K*_i_ = 106 nM; **65**, *K*_i_ = 743 nM; **68**, *K*_i_ = 263 nM and **71**, *K*_i_ = 734 nM). The observed phenomenon may arise from higher lipophilicity of the ligands, which increases non-specific interactions with the tested 5-HT_7_ receptor.

**Figure 1:**
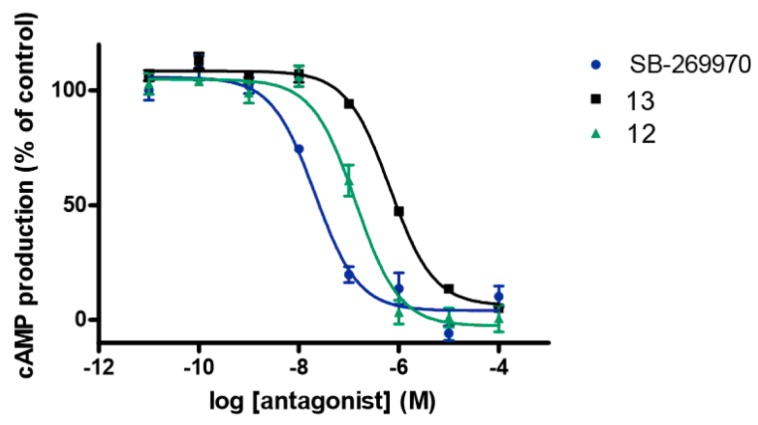
The functional properties of compounds **12** and **13** and a reference antagonist SB-269970 on 5-HT_7_R evaluated using their ability to inhibit cAMP production induced by 5-CT (10 nM) in HEK-293 cells expressing 5-HT_7_R.

Two selected compounds **12** and **13** with high 5-HT_7_R affinity were next evaluated in *in vitro* functional cAMP assay (Figure 1) and their selectivity for other serotonin (5-HT_1A_, 5-HT_2A_, 5-HT_6_) and dopamine D_2_ receptors was investigated (Table 6). It was found that both **12** and **13**, similarly to (2*R*)-1-((3-hydroxyphenyl)sulfonyl)-2-(2-(4-methyl-1-piperidinyl)ethyl)pyrrolidine (SB-269970) [32]—a selective 5-HT7R antagonist used as a control, dose-dependently decrease 5-CT-induced cAMP accumulation in transfected HEK cells expressing 5-HT_7_R (Figure 1). It has to be stressed, however, that 5-HT_7_R antagonistic properties of **12** and **13** are weaker (*K*_B_ = 12 and 60 nM, respectively) than that of SB-269970 (*K*_B_ = 1.9 nM) and that, unexpectedly, *n*-butyl derivative **13**, despite showing higher affinity, is less potent 5-HT_7_R antagonist than ethyl analog **12**. Alkyl substituent at position 4 also influences selectivity profile—both compounds show almost the same, high affinity for 5-HT_2A_R (*K*_i_ = 10 and 12 nM for **12** and **13**, respectively) but *n*-butyl derivative is 10- and 13-fold more active than **12** at 5-HT_6_ and D_2_ receptors. Both analogs are weak 5-HT_1A_R binders.

## 4. Materials and Methods

### 4.1. General Methods

All air-sensitive reactions were conducted under a nitrogen atmosphere. Tetrahydrofuran (THF) was distilled from sodium benzophenone ketyl immediately before use. Heteroaryllithium reagents were generated by direct lithiation of a heterocyclic substrate or by a bromine-lithium exchange reaction as previously reported [28] Final products were purified on a chromatotron using silica gel-coated rotors. Oily products were transformed into hydrobromide salts by using a general procedure [30] and the salts were crystallized from 95% ethanol. Melting points (Pyrex capillary) are not corrected. The ^1^H-NMR (400 MHz) and ^13^C-NMR (100 MHz) spectra of free bases and hydrobromide salts were obtained in CDCl_3_ and DMSO-*d*_6_, respectively. Mass spectra were recorded using electrospray ionization in a positive ion mode.

### 4.2. Synthesis of Substituted Pyrimidines ***1***, ***2***, ***21***, ***23***, ***24***, ***26***, ***29***, ***30***, ***32***, ***36***–***46*** and Quinazoline ***48***

A solution of the corresponding lithium reagent (10 mmol) in THF (10 mL) was stirred at −50 °C and treated dropwise with a solution of 2-chloropyrimidine, 2-chloro-5-propylpyrimidine or 2-chloroquinazoline (12 mmol) in THF (15 mL). The mixture was allowed to reach 0 °C within 2 h, then quenched with a solution of water in THF (1:5, 6 mL), stirred at 0 °C and treated with a solution of DDQ (2.3 g, 10 mmol) in THF (5 mL). After stirring for an additional 10 min at 0 °C, the mixture was treated with a cold solution of sodium hydroxide (4 M, 5 mL, 20 mmol), stirred and extracted immediately with ether/hexanes (1:1, 3 × 10 mL). The combined extracts were dried with anhydrous sodium sulfate, decolorized by filtration through a pad of silica gel (5 g) and concentrated on a rotary evaporator. The resultant crude 4-substituted 2-chloropyrimidine or 2-chloroquinazoline was treated with a primary or secondary amine (30 mmol) in toluene (20 mL) in the presence of anhydrous potassium carbonate and the mixture was heated at 75 °C for 5–10 h, after which time a TLC analysis on silica gel eluting with ether/triethylamine (9:1) showed the absence of the substrate. Preparative chromatography was conducted eluting with ether/triethylamine/hexanes (9:5:5) to give product **1**, **2**, **21**, **23**, **24**, **26**, **29**, **30**, **32**, **36**–**46** (Scheme 1) and **48** (Scheme 2).

### 4.3. Synthesis of Polysubstituted Pyrimidines ***11***–***20***, ***22***, ***25***, ***27***, ***28***, ***31***

Alternatively, the crude 4-substituted 2-chloropyrimidine, obtained as described above, was treated with a lithium reagent followed by aromatization with DDQ of the intermediate dihydropyrimidine under the conditions described above and the resultant crude 4,6-disubstituted 2-chloropyrimidine was allowed to react with *N*-methylpiperazine, as described above, to yield 4,6-disubstituted 2-(*N*-methylpiperazine-1-yl)pyrimidine **11**–**20**, **22**, **25**, **27**, **28**, **31** (Scheme 1).

### 4.4. Synthesis of [N-Substituted 2-(Piperazin-1-yl)]pyrimidines ***3***–***10*** and ***47***

In the preparation of compounds **3**–**10**, a mixture of 4-(3-furyl)-2-(piperazin-1-yl)pyrimidine (**1**, 200 mg, 0.87 mmol), synthesized as described above, and anhydrous potassium carbonate (300 mg, 2.2 mmol) in anhydrous acetonitrile (10 mL) was stirred at −10 °C and treated with ethyl bromide, propyl bromide, benzyl chloride, benzoyl chloride, 3-phenylpropyl bromide, 2-bromoethanol, ethyl 3-bromopropionate or ethyl 6-bromohexanoate (2.0 mmol) (Scheme 2). The mixture was stirred at 23 °C for 2 h and then quenched with water (2 mL) and extracted with ether (3 × 20 mL). The extract was dried with anhydrous sodium sulfate and concentrated. Purification by chromatography eluting with dichloromethane/methanol (10:1) gave product **3**–**10**.

In the preparation of 4-[4-(3-furyl)pyrimidin-2-yl]piperazine-1-spiro-1-morpholinium iodide (**47**), a mixture of compound **1** (1 mmol), 1-iodo-2-(2-iodoethoxy)ethane (1 mmol) and anhydrous potassium carbonate (140 mg, 1 mmol) in anhydrous acetonitrile (10 mL) was heated under reflux for 1 h and then quenched with water (5 mL). Product **47** was extracted with ethyl acetate and purified on a chromatotron eluting with dichloromethane/methanol (8:2).

*4-(3-Furyl)-2-(piperazin-1-yl)pyrimidine* (**1**). Yield 70% (free base); an oil; ^1^H-NMR: δ 8.33 (d, *J* = 5.1 Hz, 1H), 8.10 (s, 1H), 7.51 (m, 1H), 6.89 (m, 1H), 6.67 (d, *J* = 5.1 Hz, 1H), 3.88 (m, 4H), 2.98 (m, 4H). A hydrobromide salt: m.p. 241–242 °C. Anal. Calcd for C_12_H_14_N_4_O_2_•HBr•0.5H_2_O: C, 45.01; H, 5.03; N, 17.50. Found: C, 44.82; H, 4.85; N, 17.39.

*4-(3-Furyl)-2-(4-methylpiperazin-1-yl)pyrimidine* (**2**) [24].

*2-(4-Ethylpiperazin-1-yl)-4-(3-furyl)pyrimidine* (**3**). Yield 61% (free base); an oil; ^1^H-NMR: δ 8.29 (d, *J* = 5.1 Hz, 1H), 8.07 (m, 1H), 7.48 (m, 1H), 6.86 (m, 1H), 6.63 (d, *J* = 5.1 Hz, 1H), 3.91 (m, 4H), 2.53 (m, 4H), 2.49 (q, *J* = 7.2 Hz, 2H), 1.14 (t, *J* = 7.2 Hz, 3H). A hydrobromide salt. Anal. Calcd for C_14_H_18_N_4_O•2 HBr•0.5 H_2_O: C, 39.18; H, 4.93; N, 13.06. Found: C, 39.29; H, 4.36; N, 12.81.

*4-(3-Furyl)-2-(4-propylpiperazin-1-yl)pyrimidine* (**4**). Yield 39% (free base); an oil; ^1^H-NMR: δ 8.29 (d, *J* = 5.1 Hz, 1H), 8.06 (s, 1H), 7.48 (s, 1H), 6.86 (s, 1H), 6.63 (d, *J* = 5.1 Hz, 1H), 3.90 (t, 4H), 2.52 (m, 4H), 2.36 (t, *J* = 7.4 Hz, 2H), 1.57 (m, 2H), 0.94 (t, *J* = 7.4 Hz, 3H). A hydrobromide salt: m.p. 274–277 °C. Anal. Calcd for C_15_H_20_N_4_O•2 HBr• H_2_O: C, 39.84; H, 5.35; N, 12.39. Found: C, 40.39; H, 4.84; N, 12.45.

*2-(4-Benzylpiperazin-1-yl)-4-(3-furyl)pyrimidine* (**5**). Yield 73% (free base); an oil; ^1^H-NMR: δ 8.28 (d, *J* = 5.1 Hz, 1H), 8.05 (m, 1H), 7.47 (m, 1H), 7.31 (m, 5H), 6.85 (s, 1H), 6.62 (d, *J* = 5.1 Hz, 1H), 3.89 (m, 4H), 3.57 (s, 2H), 2.53 (m, 4H). A hydrobromide salt: m.p. >280 °C. Anal. Calcd for C_19_H_20_N_4_O•2HBr•H_2_O: C, 45.62; H, 4.84; N, 11.20. Found: C, 45.93; H, 4.67; N, 11.11.

*2-(4-Benzoylpiperazin-1-yl)-4-(3-furyl)pyrimidine* (**6**). Yield 33% (free base); an oil; ^1^H-NMR: δ 8.31 (d, *J* = 5.1 Hz, 1H), 8.06 (m, 1H), 7.48 (m, 1H), 7.44 (m, 5H), 6.85 (m, 1H), 6.69 (d, *J* = 5.1 Hz, 1H), 4.27 (broad m, 8H); ^13^C-NMR: δ 170.6, 161.7, 159.2, 158.13, 144.0, 143.1, 135.8, 129.8, 128.6, 127.1, 126.0, 108.5, 106.3, 44.0, 42.3. A hydrobromide salt: m.p. 221 °C (dec.). Anal. Calcd for C_19_H_18_N_4_O_2_•HBr•0.5 H_2_O: C, 53.79; H, 4.75; N, 13.20. Found: C, 53.97; H, 4.32; N, 13.15.

*4-(3-Furyl)-2-[(4-(3-phenylpropyl)piperazin-1-yl]pyrimidine* (**7**). Yield 44% (free base); an oil; ^1^H-NMR: δ 8.27 (d, *J* = 5 Hz, 1H), 8.05 (s, 1H), 7.46 (t, *J* = 2 Hz, 1H), 7.27 (dd, *J* = 8 Hz and 15 Hz, 2H), 7.18 (m, 3H), 6.85 (t, *J* = 2 Hz, 1H), 6.61 (d, *J* = 5 Hz, 1H), 3.88 ( t, *J* = 5 Hz, 4H), 2.67 (t, *J* = 8 Hz, 2 H), 2.50 (t, *J* = 5 Hz, 4 H), 2.41 (t, *J* = 7 Hz, 2H); ^13^C-NMR: δ 161,8, 159.0, 158.0, 143.8, 143.0, 142.1, 128.4, 128.3, 126.2, 125.7, 108.5, 105.6, 58.1, 53.1, 43.7, 33.7, 28.5. HR-MS (ESI). Calcd for C_21_H_25_N_4_O (M^+^ + 1): *m/z* 349.2028. Found: *m/z* 349.2022.

*2-[4-(2-Hydroxyethyl)piperazin-1-yl]-4-(3-furyl)pyrimidine* (**8**). Yield 58% (free base); an oil; ^1^H-NMR: δ 8.34 (t, *J* = 4.8 Hz, 1H), 8.27 (d, *J* = 5.2 Hz, 1H), 7.47 (d, *J* = 1.2 Hz, 1H), 6.85 (s, 1H), 6.62 (d, *J* = 5.2 Hz, 1H), 3.89 (t, *J* = 4.8 Hz, 4H), 3.68 (t, *J* = 5.2 Hz, 2H), 3.20 (broad s, exchangeable with D_2_O, 1H), 2.58 (m, 6H); ^13^C-NMR: δ 161.9, 159.2, 158.2, 143.3, 143.1, 126.3, 108.7, 105.9, 59.8, 58.0, 53.0, 43.8. HR-MS (ESI). Calcd for C_14_H_19_N_4_O_2_ (M^+^ + 1): *m/z* 275.1508. Found: *m/z* 275.1508. A hydrobromide salt: m.p. >280 °C. Anal. Calcd for C_14_H_19_N_4_O_2_•2HBr•H_2_O: C, 37.02; H, 4.88; N, 12.34. Found: C, 37.12; H, 4.51; N, 12.32.

*Ethyl 3-{4-{[4-(3-furyl)pyrimidin-2-yl]piperazin-1-yl}}propanoate* (**9**). Yield 50% (free base); an oil; ^1^H-NMR: δ 8.27 (d, *J* = 5 Hz, 1H), 8.06 (s, 1H), 7.47 (t, *J* = 2 Hz, 1H), 6.85 (m, 1H), 6.62 (d, *J* = 5 Hz, 1H), 4.15 (q, *J* = 7 Hz, 2H), 3.87 (t, *J* = 5 Hz, 4H), 2.74 (m, 2H), 2.54 (m, 6H), 1.26 (t, *J* = 7 Hz, 3H); ^13^C-NMR: δ 172.6, 161.9, 159.1, 158.1, 144.0, 143.1, 126.3, 108.6, 105.8, 60.6, 53.8, 53.0, 43.8, 32.5, 14.4. HR-MS (ESI). Calcd for C_17_H_23_N_4_O_3_ (M^+^ + 1): *m/z* 331.1770. Found: *m/z* 331.1756.

*Ethyl 3-{4-{[4-(3-furyl)pyrimidin-2-yl]piperazin-1-yl}}hexanoate* (**10**). Yield 85% (free base); an oil; ^1^H-NMR: δ 8.27 (d, *J* = 5 Hz, 1H), 8.06 (s, 1H), 7.48 (t, *J* = 2 Hz, 1H), 6.85 (m, 1H), 6.62 (d, *J* = 5 Hz, 1H), 4.12 (q, *J* = 7 Hz, 2H), 3.88 (t, *J* = 5 Hz, 4H), 2.50 (m, 4H), 2.30 (t, *J* = 8 Hz, 2H), 2.31 (t, *J* = 8 Hz, 2H), 1.66 (m, 2H), 1.56 (m, 2H), 1.37 (m, 2H), 1.25 (t, *J* = 7 Hz, 3H); ^13^C-NMR: δ 173.7, 161.7, 158.9, 158.0, 143.8, 143.0, 108.5, 105.6, 60.2, 58.6, 53.2, 43.6, 34.2, 27.1, 26.5, 24.9, 14.2. HR-MS (ESI). Calcd for C_20_H_29_N_4_O_3_ (M^+^ + 1): *m*/*z* 373.2240. Found: m/z 373.2240.

*4-(3-Furyl)-6-methyl-2-(4-methylpiperazin-1-yl)pyrimidine* (**11**) [24].

*4-Ethyl-6-(3-furyl)-2-(4-methylpiperazin-1-yl)pyrimidine* (**12**). Yield 67% (free base); an oil; ^1^H-NMR: δ 8.06 (m, 1H), 7.50 (m, 1H), 6.88 (m, 1H), 6.56 (s, 1H), 3.94 (m, 4H), 2.65 (d, *J* = 7.5 Hz, 2H), 2.51 (m, 4H), 2.38 (s, 3H), 1.33 (t, *J* = 7.5 Hz, 3H). A hydrobromide salt: m.p. 287-288 °C. Anal. Calcd for C_15_H_20_N_4_O•2HBr•0.5H_2_O: C, 40.47; H, 5.66; N, 12.58. Found: C, 40.41; H, 5.32; N, 12.55.

*4-(n-Butyl)-6-(3-furyl)-2-(4-methylpiperazin-1-yl)pyrimidine* (**13**). Yield 30% (free base); an oil; ^1^H-NMR: δ 0.91 (m, 3H), 1.35 (m, 2H), 1.73 (m, 2H), 2.35 (s, 3H), 2.47 (t, *J* = 5 Hz, 4H), 2.58 (t, *J* = 7 Hz, 2H), 3.90 (t, *J* = 5 Hz, 4H), 6.50 (s, 1H), 6.85 (m, 1H), 7.46 (m, 1H), 8.06 (m, 1H). A hydrobromide salt: m.p. 179–180 °C. Anal. Calcd for C_17_H_24_N_4_O•2HBr•1.5H_2_O: C, 43.89; H, 6.28; N, 12.04. Found: C, 43.95; H, 6.30; N, 11.96.

*4-(3-Furyl)-6-n-hexyl-2-(4-methylpiperazin-1-yl)pyrimidine* (**14**). Yield 51% (free base); an oil; ^1^H-NMR: δ 0.90 (m, 3H), 1.32 (m, 6H), 1.70 (m, 2H), 2.34 (s, 3H), 2.47 (t, *J* = 5 Hz, 4H), 2.57 (t, *J* = 7.5 Hz, 2H), 3.91 (t, *J* = 5 Hz, 4H), 6.51 (s, 1H), 6.85 (m, 1H), 7.46 (m, 1H), 8.06 (m, 1H); ^13^C-NMR: δ 14.1, 22.6, 28.5, 29.1, 31.7, 38.1, 43.6, 46.3, 55.1, 104.6, 108.6, 126.4, 142.7, 143.7, 158.6, 162.0, 171.8. A hydrobromide salt: m.p. 230–233 °C. Anal. Calcd for C_19_H_28_N_4_O•HBr•0.5H_2_O: C, 54.54; H, 7.23; N, 13.39. Found: C, 54.34; H, 7.06; N, 13.29.

*4-sec-Butyl-6-(3-furyl)-2-(4-methylpiperazin-1-yl)pyrimidine* (**15**). Yield 37% (free base); an oil; ^1^H-NMR: δ 0.87 (t, *J* = 7.5 Hz, 3H), 1.25 (m, 3H), 1.57 (m, 1H), 1.76 (m, 1H), 2.34 (s, 3H), 2.47 (t, *J* = 5 Hz), 2.57 (m, 1H), 2.90 (t, *J* = 5 Hz, 4H), 6.49 (s, 1H), 6.87 (m, 1H), 7.47 (m, 1H), 8.06 (m, 1H); ^13^C-NMR: δ 12.1, 19.6, 29.1, 43.4, 43.7, 46.3, 55.1, 103.7, 108.6, 126.5, 142.7, 143.6, 158.6, 162.0, 175.8. A dihydrobromide salt: m.p. 165–170 °C. Anal. Calcd for C_17_H_24_N_4_O•2HBr•0.5H_2_O: C, 43.33; H, 5.77; N, 11.89. Found: C, 43.05; H, 5.64; N, 11.88.

*4-(3-Furyl)-2-(4-methylpiperazin-1-yl)-6-(trifluoromethyl)pyrimidine* (**16**). Yield 81% (free base); an oil. A hydrochloride salt: m.p. >300 °C; ^1^H-NMR: δ 8.77 (s, 1H), 7.88 (m, 1H), 7.56 (m, 1H), 3.98 (m, 4H), 2.55 (m, 4H), 2.39 (s, 3H). Anal. Calcd for C_14_H_15_N_4_F_3_O•HCl•0.25H_2_O: C, 47.00; H, 4.71; N, 15.86. Found: C, 47.40; H, 4.61; N, 15.72.

*4-(Cyclohexenylethynyl)-6-(3-furyl)-2-(4-methylpiperazin-1-yl)pyrimidine* (**17**). Yield 8%; a colorless oil (free base); ^1^H-NMR: 1.68 (m, 4H, CH_2_), 2.18 (m, 2H), 2.27 (m, 2H), 2.34 (s, 3H, CH_3_), 2.48 (t, *J* = 5 Hz, 4H), 3.90 (t, *J* = 5 Hz, 4H), 6.39 (m, 1H), 6.74 (s, 1H), 6.86 (m, 1H), 7.49 (m, 1H), 8.07 (m, 1H); ^13^C-NMR: δ 21.3, 22.2, 25.9, 28.7, 43.6, 46.2, 55.0, 85.7, 93.2, 108.3, 108.5, 119.9, 125.9, 138.5, 143.1, 143.9, 151,8, 159.1, 161.8. A dihydrobromide salt: m.p. 188–190 °C. Anal. Calcd for C_21_H_24_N_4_O•2HBr•1.75H_2_O: C, 46.55; H, 5.49; N, 10.34. Found: C, 46.63; H, 5.09; N, 10.00.

*4,6-Bis(3-furyl)-2-(4-methylpiperazin-1-yl)pyrimidine* (**18**) [24].

### 4.5. Synthesis of 4-Dimethylamino-6-(3-furyl)-2-(4-methylpiperazin-1-yl)pyrimidine *(**19**)*

This compound was obtained by: (1) addition reaction of 3-furyllithium with 2,4-dichloropyrimidine; (2) aromatization of the intermediate dihydropyrimidine with DDQ by using the general procedure described above; (3) treatment of the crude product [2,4-dichloro-6-(3-furyl)pyrimidine] with dimethylamine in ethanol at 23 °C, which caused a selective displacement of the 4-chloro atom to yield 2-chloro-4-dimethylamino-6-(3-furyl)pyrimidine; and (4) the treatment of this product with 4-methylpiperazine at elevated temperature using the general procedure described above.

Yield 30%; a colorless oil (free base); ^1^H-NMR: δ 8.17 (m, 1H), 7.90 (m, 1H), 7.48 (m, 1H), 6.95 (s, 1H), 3.86 (m, 4H), 2.48 (m, 4H), 2.35 (s, 3H), 2.20 (s, 6H); ^1^H-NMR NOE experiment: Irradiation at δ 2.20 (NMe_2_) gave a strong NOE signal at δ 6.95 for C5-H of the pyrimidine, which is fully consistent with the given structure. HR-MS (ESI). Calcd for C_15_H_21_N_5_O: *m/z* 287.1745. Found: *m/z* 2871748.

*4-(3-Furyl)-2,4-bis(4-methylpiperazin-1-yl)pyrimidine* (**20**)

This compound was obtained by treatment of 2,4-dichloro-6-(furan-3-yl)pyrimidine described above with 4-methylpiperazine at 75 °C by using the general procedure described above.

Yield 37% (free base); an oil; ^1^H-NMR: δ 7.47 (m, 1H), 7.05 (m, 1H), 6.53 (m, 1H), 6.31 (s, 1H), 3.85 (t, *J* = 4.8 Hz, 4H), 3.67 (t, *J* = 4.8 Hz, 4H), 2.47 (t, *J* = 4.8 Hz, 8H), 2.34 (s, 6H); ^13^C-NMR: δ 163.4, 161.7, 155.4, 153.8, 143.2, 111.8, 109.7, 86.9, 55.2, 54.8, 46.3, 46.2, 43.9, 43.8. Anal. Calcd for C_18_H_26_N_6_O•3HBr: C, 36.94; H, 5.00; N, 14.36. Found: C, 37.14; H, 5.29; N, 14.62.

*4-(3-Furyl)-2-(4-methylpiperazino)-5-propylpyrimidine* (**21**). Yield 44%; an oil; ^1^H-NMR (free base): δ 8.17 (m, 1H), 7.91 (m, 1H), 7.48 (m, 1H), 6.95 (s, 1H), 3.85 (m, 4H), 2.59 (t, *J* = 7.6 Hz, 2H), 2.49 (m, 4H), 2.35 (s, 3H), 1.59 (sxt, *J* = 7.6 Hz, 2H), 0.99 (t, *J* = 7.6 Hz, 3H). A hydrobromide salt: m.p. 206–208 °C. Anal. Calcd for C_16_H_22_N_4_O•HBr: C, 52.32; H, 6.31; N, 15.25. Found: C, 52.34; H, 6.11; N, 15.12.

*4-n-Butyl-6-(3-furyl)-2-(4-methylpiperazino)-5-propylpyrimidine* (**22**). Yield 73% (free base); an oil; ^1^H-NMR: δ 7.80 (m, 1H), 7.47 (m, 1H), 6.86 (m, 1H), 3.84 (m, 4H), 2.66 (m, 2H), 2.59 (m, 2H), 2.47 (m, 4H), 2.34 (s, 3H), 1.69 (m, 2H), 1.47 (m, 4H), 1.01 (t, *J* = 7.6 Hz, 3H), 0.96 (t, *J* = 7.6 Hz, 3H). A dihydrobromide salt: m.p. 203 °C (dec.). Anal. Calcd for C_20_H_30_N_4_O•2HBr: C, 47.63; H, 6.40; N, 11.11. Found: C, 47.34; H, 6.65; N, 11.08.

*4-(2-Methyl-3-furyl)-2-(4-methylpiperazin-1-yl)pyrimidine* (**23**). Yield 39% (free base); an oil. A dihydrobromide salt: m.p. 263–265 °C; ^1^H-NMR: δ 2.66 (s, 3H, CH_3_), 2.85 (s, 3H), 3.11 (m, 2H), 3.40 (m, 2H, CH_2_), 3.55 (m, 2H), 4.73 (m, 2H), 6.96 (m, 1H), 6.98 (m, 1H), 7.59 (m, 1H), 8.41 (m, 1H), 10.00 (bs, 1H, exchangeable with D_2_O). Anal. Calcd for C_14_H_18_N_4_O•2HBr•0.5 H_2_O: C, 39.18; H, 4.93; N, 13.06. Found: C, 39.18; H, 4.77; N, 13.32.

*4-(5-Methyl-3-furyl)-2-(4-methylpiperazin-1-yl)pyrimidine* (**24**). Yield 58% (free base); an oil. A dihydrobromide salt: m.p. 275 °C (dec.); ^1^H-NMR: δ 2.30 (s, 3H, CH_3_), 2.84 (m, 3H, CH_3_), 3.10 (m, 2H), 3.31 (m, 2H), 3.53 (m, 2H), 4.79 (m, 2H), 6.72 (m, 1H), 7.04 (m, 1H), 8.41 (m, 2H), 9.98 (bs, 1H, exangeable with D_2_O). Anal. Calcd for C_14_H_18_N_4_O•2HBr•0.5 H_2_O: C, 39.18; H, 4.93; N, 13.06. Found: C, 39.01; H, 4.70; N, 13.02.

*4-n-Hexyl-6-(5-methyl-3-furyl)-2-(4-methylpiperazin-1-yl)pyrimidine* (**25**). Yield 22% (free base); an oil. A dihydrobromide salt: m.p. 242 °C (dec.); ^1^H-NMR: δ 0.88 (m, 3H), 1.34 (m, 6H), 1.69 (m, 2H), 2.31 (s, 3H), 2.57 (t, *J* = 4.5 Hz , 2H), 2.86 (m, 3H), 3.10 (m, 2H), 3.26 (m, 2H), 3.50 (m, 2H), 4.81 (m, 2H), 6.67 (s, 1H), 6.91 (s, 1H), 8.28 (s, 1H), 9.37 (bs, 1H, exchangeable with D_2_O). Anal. Calcd for C_20_H_30_N_4_O•2HBr•H_2_O: C, 45.99; H, 6.56; N, 10.73. Found: C, 45.81; H, 6.44; N, 10.54.

*4-(2-Furyl)-2-(4-methylpiperazin-1-yl)pyrimidine* (**26**) [29].

*4-Ethyl-6-(2-furyl)-2-(4-methylpiperazin-1-yl)pyrimidine* (**27**). Yield 61% (free base); an oil; ^1^H-NMR: δ 7.52 (m, 1H), 7.12 (d, *J* = 3.2 Hz, 1H), 6.77 (s, 1H), 6.51 (m, 1H), 3.91 (t, *J* = 4.8 Hz, 4H), 2.64 (q, *J* = 7.4 Hz, 2H), 2.48 (t, *J* = 4.8 Hz, 4H), 2.34 (s, 3H), 1.27 (t, *J* = 7.4 Hz, 3H); ^13^C-NMR: δ 173.2, 162.0, 155.8, 153.3, 144.1, 112.1, 110.8, 102.4, 55.3, 46.5, 43.8, 31.4, 12.7. HR-MS (ESI). Calcd for C_15_H_21_N_4_O (M^+^ + 1): *m/z* 273.1725. Found: *m/z* 273.1715. A hydrobromide salt: m.p. >250 °C. Anal. Calcd for C_15_H_20_N_4_O•2HBr: C, 41.50; H, 5.11; N, 12.70. Found: C, 41.79; H, 4.87; N, 12.38.

*4-n-Butyl-6-(2-furyl)-2-(4-methylpiperazin-1-yl)pyrimidine* (**28**). Yield 78% (free base); an oil; ^1^H-NMR: δ 7.52 (m, 1H), 7.11 (d, *J* = 3.2 Hz, 1H), 6.75 (s, 1H), 6.51 (m, 1H), 3.90 (t, *J* = 4.6 Hz, 4H), 2.60 (t, *J* = 7.4 Hz, 2H), 2.48 (t, *J* = 4.6 Hz, 4H), 2.34 (s, 3H), 1.70 (m, 2H), 1.40 (m, 2H), 0.94 (t, J = 7.4 Hz, 3H); ^13^C-NMR: δ 172.4, 162.1, 155.8, 153.4, 144.1, 112.2, 110.8, 103.1, 55.3, 46.5, 43.9, 38.1, 30.8, 22.7, 14.2. HR-MS (ESI). Calcd for C_17_H_25_N_4_O (M^+^ + 1): *m/z* 301.2026. Found: *m/z* 301.2028. Anal. Calcd for C_17_H_24_N_4_O•HBr: C, 53.55; H, 6.61; N, 14.10. Found: C, 53.34; H, 6.60; N, 14.49.

*4-(5-Methyl-2-furyl)-2-(4-methylpiperazin-1-yl)pyrimidine* (**29**). Yield 48% (free base); an oil. A dihydrobromide salt: m.p. 263–265 °C; ^1^H-NMR: δ 2.39 (s, 3H), 2.85 (m, 3H), 3.11 (m, 2H), 3.28 (m), 3.54 (m, 2H), 4.79 (m, 2H), 6.36 (m, 1H), 6.99 (m, 1H), 7.28 (m, 1H), 8.45 (m, 1H), 9.78 (bs, 1H, exchangeable with D_2_O). Anal. Calcd for C_14_H_18_N_4_O•2HBr: C, 40.02; H, 4.80; N, 13.34. Found: C, 39.70; H, 4.85; N, 13.25.

*2-(Methylpiperazin-1-yl)-4-(3-thienyl)pyrimidine* (**30**) [25].

*4-n-Butyl-2-(methylpiperazin-1-yl)-6-(3-thienyl)pyrimidine* (**31**) [30].

*2-(Methylpiperazin-1-yl)-4-(2-thienyl)pyrimidine* (**32**) [24,26].

*4-(2-Hydroxyphenyl)-2-(4-methylpiperazin-1-yl)pyrimidine* (**33**) [27].

*4-(3-Hydroxyphenyl)-2-(4-methylpiperazin-1-yl)pyrimidine* (**34**) [27].

*4-(4-Hydroxyphenyl)-2-(4-methylpiperazin-1-yl)pyrimidine* (**35**) [27].

*4-(Benzofuran-2-yl)-2-(4-methylpiperazin-1-yl)pyrimidine* (**36**). Yield 71% (free base); an oil; ^1^H-NMR: δ 8.45 (m, 1H), 7.69 (m, 1H), 7.58 (m, 1H), 7.42 (m, 1H), 7.30 (m, 2H), 7.09 (m, 1H), 3.98 (m, 4H), 2.55 (m, 4H, 2.39 (s, 3H). A hydrobromide salt: m.p. >300 °C. Anal. Calcd for C_17_H_18_N_4_O•2HBr: C, 44.76; H, 4.42; N, 12.28. Found: C, 44.60; H, 4.48; N, 12.15.

*4-(Benzothiophen-2-yl)-2-(4-methylpiperazin-1-yl)pyrimidine* (**37**). Yield 78% (free base); an oil; ^1^H-NMR: δ 8.39 (d, *J* = 5.1 Hz, 1H), 7.96 (s, 1H), 7.88 (m, 2H), 7.41 (m, 2H), 6.99 (d, *J* = 5.1 Hz, 1H), 3.98 (m, 4H), 2.55 (m, 4H), 2.40 (s, 3H). A hydrobromide salt: m.p. 299–300 °C. Anal. Calcd for C_17_H_18_N_4_S•2HBr: C, 43.24; H, 4.27; N, 11.86. Found: C, 43.20; H, 4.33; N, 11.80.

*4-(1-Methylindol-2-yl)-2-(4-methylpiperazin-1-yl)pyrimidine* (**38**) [27].

*2-(4-Methylpiperazin-1-yl)-4-(pyridin-3-yl)pyrimidine* (**39**) [27].

*2-(4-Methylpiperazin-1-yl)-4-(thiazol-2-yl)pyrimidine* (**40**). Yield 77% (free base); an oil; ^1^H-NMR: δ 8.44 (d, *J* = 5.0 Hz, 1H), 7.95 (d, *J* = 3.2 Hz, 1H), 7.48 (d, *J* = 3.2 Hz, 1H), 7.29 (d, *J* = 5.0 Hz, 1H), 3.92 (t, *J* = 5.0 Hz, 4H), 2.50 (t, *J* = 5.0 Hz, 4H), 2.36 (s, 3H); ^13^C-NMR: δ 168.4, 161.4, 159.0, 157.7, 144.3, 122.1, 104.3, 54.8, 46.1, 43.5. A hydrobromide salt: m.p. 273 °C (decomp.). Anal. Calcd for C_12_H_15_N_5_S•HBr•0.5H_2_O: C, 41.03; H, 4.88; N, 19.94. Found: C, 41.32; H, 4.64; N, 19.93.

*(1H-Imidazol-1-yl)-2-(4-methylpiperazin-1-yl)pyrimidine* (**41**). Yield 87% (free base); an oil; ^1^H-NMR: δ 8.36 (s, 1H), 8.36 (d, *J* = 5.4 Hz, 1H), 7.60 (s, 1H), 7.17 (s, 1H), 6.50 (d, *J* = 5.4 Hz, 1H), 3.90 (t, *J* = 5.1 Hz, 4H), 2.49 (t, *J* = 5.1 Hz, 4H), 2.35 (s, 3H); ^13^C-NMR: δ 161.4, 160.1, 155.3, 135.0, 130.8, 115.6, 96.5, 54.8, 46.1, 43.7. A hydrobromide salt: m.p. 229–231 °C. Anal. Calcd for C_12_H_16_•2HBr•H_2_O: C, 33.98; H, 4.75; N, 19.81. Found: C, 33.93; H, 4.55; N, 19.60.

*4-(3-Furyl)-2-(4-methylhomopiperazin-1-yl)pyrimidine* (**42**). Yield 69% (free base); an oil; ^1^H-NMR: δ 8,32 (d, *J* = 5.1 Hz, 1H), 8.08 (s, 1H), 7.50 (m, 1H), 6.88 (m, 1H), 6.64 (d, *J* = 5.1 Hz, 1H), 4.01 (m, 2H), 3.89 (m, 2H), 2.74 (m, 2H), 2.60 (m, 2H), 2.41 (s, 3H), 2.04 (m, 2H). A hydrobromide salt: m.p. 176–177 °C. Anal. Calcd for C_14_H_18_N_4_O•2HBr•1.25H_2_0: C, 37.99; H, 5.12; N, 12.66. Found: C, 37.96; H, 5.12; N, 12.51.

*4-(3-Furyl)-2-(piperidin-1-yl)pyrimidine* (**43**). Yield 68% (free base); an oil; ^1^H-NMR: δ 8.32 (d, *J* = 5.1 Hz, 1H), 8.10 (m, 1H), 7.51(m, 1H), 6.90 (m, 1H), 6.62 (d, *J* = 5.1 Hz, 1H), 3.89 (m, 4H), 1.67 (m, 6H). A hydrobromide salt: m.p. 190–191 °C. Anal. Calcd for C_13_H_15_N_3_O•HBr•0.25H_2_O: C, 49.66; H, 5.28; N, 13.35. Found: C, 49.60; H, 5.11, N, 13.37.

*4-(4-(3-Furyl)-2-(morpholin-1-yl)pyrimidine* (**44**). Yield 53% (free base); a white solid: m.p. 58–59 °C; ^1^HNMR: δ 3.79 (m, 4H), 3.87 (m, 4H), 6.68 (d, *J* = 4.8 Hz, 1H), 6.88 (m, 1H), 7.49 (m, 1H), 8.08 (m, 1H), 8.31 (d, *J* = 4.8 Hz, 1H). A hydrobromide salt: m.p. 215-216 °C; Anal. Calcd for C_12_H_13_N_3_O_2_•HBr•0.25 H_2_O: C, 45.51; H, 4.62; N, 13.27. Found: C, 45.38; H, 4.52; N, 13.37.

*4-(3-Furyl)-N-[2-(morpholin-4-yl)ethyl]pyrimidin-2-amine* (**45**). Yield 69% (free base); an oil; ^1^H-NMR: δ 8.25 (d, *J* = 5.2 Hz, 1H), 8.07 (s, 1H), 7.48 (d, *J* = 1.8 Hz, 1H), 6.85 (m, 1H), 6.66 (d, *J* = 5.2 Hz, 1H), 5.78 (s, 1H), 3.71 (t, *J* = 4.4 Hz, 4H), 3.55 (q, *J* = 6 Hz, 2H),2.59 (t, *J* = 6 Hz, 2H), 2.49 (t, *J* = 4.4 Hz, 4H). A hydrobromide salt: m.p. 143–146 °C. Anal. Calcd for C_14_H_18_N_4_O_2_•2HBr•0.5H_2_0: C, 37.77; H, 4.75; N, 12.59. Found: C, 37.78; H, 4.76; N, 12.20.

*4-(3-Furyl)-N-[3-(1H-imidazol-2-yl)propyl]pyrimidin-2-amine* (**46**). Yield 62% (free base); an oil; ^1^H-NMR: δ 8.24 (d, *J* = 5.2 Hz, 1H), 8.06 (m, 1H), 7.49 (m, 2H), 7.06 (s, 1H), 6.94 (s, 1H), 6.84 (m, 1H), 6.70 (d, *J* = 5.2 Hz, 1H), 4.04 (t, *J* = 6.8 Hz, 2H), 3.46 (t, *J* = 6.8 Hz, 2H), 2.09 (m, 2H). A hydrobromide salt: m.p. 234–236 °C. Anal. Calcd for C_14_H_15_N_5_O•2HBr•0.5H_2_O: C, 38.30; H, 4.12; N, 15.91. Found: C, 38.23; H, 3.93; N, 15.78.

### 4.6. Synthesis of 4-[4-(3-Furyl)pyrimidin-2-yl]piperazine-1-spiro-1-morpholinium iodide (***47***)

A mixture of compound **1** (1 mmol), 1-iodo-2-(2-iodoethoxy)ethane (1 mmol) and anhydrous potassium carbonate (140 mg, 1 mmol) in anhydrous acetonitrile (10 mL) was heated under reflux for 1 h and then quenched with water (5 mL). Product **47** was extracted with ethyl acetate and purified on a chromatotron eluting with dichloromethane/methanol (8:2).

Yield 66%; m.p. >200 °C (decomp.); ^1^H-NMR (DMSO-*d*_6_): δ 8.55 (m, 1H), 8.46 (d, *J* = 5.2 Hz, 1H), 7.83 (m, 1H), 7.10 (m, 2H), 4.13 (m, 4H), 3.98 (m, 4H), 3.74 (m, 4H), 3.67 (m, 4H). HR-MS (ESI). Calcd for C_16_H_21_N_4_O_2_ (M^+^): *m/z* 301.1665. Found: *m/z* 301.1650. Anal. Calcd for C_16_H_21_N_4_IO_2_: C, 44.87; H, 4.94; N, 13.08. Found: C, 44.97; H, 5.09; N, 12.69.

*4-(3-Furyl)-2-(methylpiperazin-1-yl)quinazoline* (**48**) [27].

### 4.7. Synthesis of 2,4-bis(4-Methylpiperazin-1-yl)quinazoline (***49***)

This compound was obtained by treatment of 2,4-dichloroquinazoline with 4-methylpiperazine by using the general necleophilic displacement procedure described above.

Yield 46% (free base); an oil; ^1^H-NMR: δ 7.67 (d, *J* = 8.4 Hz, 1H), 7.51 (m, 2H), 7.05 (m, 1H), 3.93 (m, 4H), 3.70 (t, *J* = 4.6 Hz, 4H), 2.61 (t, *J* = 4.6 Hz, 4H), 2.51 (t, *J* = 4.6 Hz, 4H), 2.37 2s, 6H); ^13^C-NMR: δ 165.8, 158.4, 154.6, 132.6, 126.4, 125.3, 120.8, 112.2, 55.3, 55.1, 49.9, 46.4, 46.3, 44.1. HR-MS (ESI). Calcd for C_18_H_27_N_6_ (M^+^ + 1): *m/z* 327.2282. Found: *m/z* 327.2297. A hydrobromide salt: m.p. 108–112 °C. Anal. Calcd for C_18_H_26_N_6_•2HBr: C, 44.28; H 5.78; N, 17.21. Found: C, 44.60; H, 5.73; N, 17.42.

### 4.8. Synthesis of 4-[2-(Dimethylamino)ethyl]-2-[2-(4-methylpiperazin-1-yl)quinazoline (***50***)

The starting material, 2-chloro-4-vinylquinazoline, was obtained by the reaction of 2-chloroquinazoline with vinyllithium followed by aromatization of the resultant 4-vinyldihydroquinazoline adduct by treatment with potassium ferricyanide(III) according to the described procedure [36].

The conjugate addition reaction of dimethylamine with 2-chloro-4-vinylquinazoline to give 2-chloro-4-(2-dimethylaminoethyl)quinazoline was conducted as described for the preparation of analogous compounds [27]. Treatment of this compound with 4-methylpiperazine to give the final product **50** was conducted by using a general procedure described above.

Yield 27% (free base), an oil; ^1^H-NMR: δ 7.86 (d, *J* = 8.0 Hz, 1H), 7.59 (m, 2H), 7.18 (t, *J* = 7.2 Hz, 1H), 3.99 (t, *J* = 5.0 Hz, 4H), 3.29 (t, *J* = 7.2 Hz, 2H), 2.85 (t, *J* = 7.2 Hz, 2H), 2.51 (t, *J* = 5.0 Hz, 4H), 2.36 (s, 6H), 2.35 (s, 3H); ^13^C-NMR: δ 170.1, 158.4, 152.3, 133.3, 126.4, 124.6, 122.1, 118.6, 57.6, 55.1, 46.3, 45.5, 43.8, 32.4. HR-MS (ESI). Calcd for C_17_H_26_N_5_ (M^+^ + 1): *m/z* 300.2175. Found: *m/z* 300.2188. A hydrobromide salt: m.p. 100–106 °C. Anal. Calcd for C_17_H_25_N_5_•3.5HBr•H_2_O: C, 34.00; H, 5.12; N, 11.66. Found: C, 33.74; H, 5.40; N, 11.26. 

### 4.9. Synthesis of 2-(4-Methylpiperazin-1-yl)pyridines ***51**–**53***

A mixture of furan-3-boronic acid, furan-2-boronic acid or thiophene-2-boronic acid (3.4 mmol), 4-bromo-2-chloropyrimidine (0.25 mL, 2.3 mmol), potassium carbonate (1.0 g, 7 mmol) and tetrakis(triphenylphosphine)palladium [Pd(PPh3)4, 0.2 g, 0.2 mmol] in anhydrous dioxane (6 mL) was stirred under a nitrogen atmosphere and heated under reflux for 4 days. After cooling the mixture was filtered through a 0.5-cm pad of Celite and concentrated under reduced pressure. A mixture of the crude product (2-chloro-4-heteroarylpyridine, 0.5 mmol) and 1-methylpiperazine (1 mL) in a sealed tube was heated at 200 °C for 12 h. The resultant brown oil was subjected to chromatography eluting with dichloromethane/methanol (95:5) to yield analytically pure product **51**–**53**.

*4-(3-Furyl)-2-(4-methylpiperazin-1-yl)pyridine* (**51**). Yield 20% (free base); an oil; ^1^H-NMR: δ 8.16 (m, 1H), 7.81 (s, 1H), 7.49 (m, 1H), 6.72 (m, 3H), 3.60 (t, *J* = 5.0 Hz, 4H), 2.53 (t, *J* = 5.0 Hz, 4H), 2.34 (s, 3H); ^13^C-NMR: δ 160,1, 148.4, 144.0, 141.4, 140.0, 125.2, 110.9, 108.5, 103.5, 54.9, 46.1, 45.2. A hydrobromide salt: m.p. >250 °C. Anal. Calcd for C_14_H_17_N_3_0•2HBr: C, 41.51; H, 4.73; N, 10.37. Found: C, 41.14; H, 4.39; N, 11.07.

*4-(2-Furyl)-2-(4-methylpiperazin-1-yl)pyridine* (**52**)*.* Yield 55% (free base); an oil; ^1^H-NMR: δ 8.17 (m, 1H), 7.49 (m, 1H), 6.39 (s, 1H), 6.86 (m, 1H), 6.78 (m, 1H), 6.49(m, 1H), 3.62 (t, *J* = 5.0 Hz, 4H), 2.54 (t, *J* = 5.0 Hz, 4H), 2.35 (s, 3H); ^13^C-NMR: δ 160.1, 152.3, 148.3, 143.1, 138.8, 111.9, 108.4, 107.9, 100.9, 54.9, 46.2, 45.2. HR-MS (ESI). Calcd for C_14_H_18_N_3_O (M^+^ + 1): *m/z* 244.1450. Found: *m/z* 244.1459. A hydrobromide salt: m.p. >250 °C. Anal. Calcd for C_14_H_17_N_3_O•2HBr: C, 41.51; H, 4.73; N, 10.37. Found: C, 41.24; H, 4.78; N, 10.51.

*2-(4-Methylpiperazin-1-yl)-4-(2-thienyl)pyridine* (**53**)*.* Yield 34% (free base); an oil; ^1^H-NMR: δ 8.18 (d, *J* = 5.2 Hz, 1H), 7.44 (m, 1H), 7.36 (d, *J* = 4.5 Hz, 1H), 7.11 (m, 1H), 6.86 (m, 1H), 6.83 (s, 1H), 3.62 (t, *J* = 5.0 Hz, 4H), 2.55 (t, *J* = 5.0 Hz, 4H), 2.36 (s, 3H). ^13^C-NMR: δ 160.1, 148.5, 142.8, 142.5, 128.1, 126.3, 124.8, 110.7, 103.2, 54.9, 46.2, 45.3. A hydrobromide salt: m.p. >250 °C. Anal. Calcd for C_14_H_17_N_3_S•2HBr•0.5H_2_0: C, 39.09; H, 4.69; N, 9.77. Found: C, 39.46; H, 4.80; N, 9.44.

### 4.10. Synthesis of Pyridines ***54**–**59***

A mixture of 4-bromo-6-methyl-2-(methylpiperazin-1-yl)pyridine or 4-bromo-2-(methylpiperazin-1-yl)pyridine (0.4 mmol), furan-3-boronic acid, furan-2-boronic acid or thiophene-2-boronic acid (0.5 mmol), tetrakis(triphenylphosphine)palladium (0.03 g, 0.03 mmol) and potassium carbonate 0.17 g, 1.1 mmol) in DMF (4.0 mL) containing water (0.3 mL) in a sealed tube under a nitrogen atmosphere was stirred at 90 °C for 12 h. After cooling, the mixture was filtered through Celite and dissolved in ethyl acetate (20 mL). The solution was washed with water (3 × 25 mL), brine (20 mL) and concentrated under reduced pressure. The residue was subjected to chromatography eluting with hexanes/ether (4:1, 200 mL; 1:1, 150 mL) and finally with hexanes/ether/methanol (10:10:1, 200 mL) to give product **54**–**59**.

*4-(3-Furyl)-2-methyl-6-(4-methylpiperazin-1-yl)pyridine* (**54**). Yield 31% (free base); an oil; ^1^H-NMR: δ 7.79 (s, 1H), 7.48 (s, 1H), 6.69 (s, 1H), 6.62 (s, 1H), 6.61 (s, 1H), 3.60 (t, *J* = 4.8 Hz, 4H), 2.55 (t, *J* = 4.8 Hz, 4H),2.42 (s, 3H), 2.36 (s, 3H); ^13^C-NMR: δ 157.5, 144.0, 141.8, 140.0, 128.7, 125.6, 110.5, 108.8, 100.6, 55.1, 46.4, 45.5, 24.8. HR-MS (ESI). Calcd for C_15_H_20_N_2_O (M^+^ + 1): *m/z* 258.1606. Found: *m/z* 258.1598. A hydrobromide salt: m.p. 150–152 °C. Anal. Calcd for C_15_H_19_N_3_O•HBr•0.5H_2_0: C, 51.88; H, 6.10; N, 12.10. Found: C, 51.90; H, 6.50; N, 12.06.

*4-(2-Furyl)-2-methyl-6-(4-methylpiperazin-1-yl)pyridine* (**55**). Yield 10% (free base); an oil; ^1^H-NMR: δ 7.47 (s, 1H), 6.75 (m, 2H), 6.72 (s, 1H), 6.47 (m, 1H), 3.62 (t, *J* = 4.8 Hz, 4H), 2.54 (t, *J* = 4.8 Hz, 4H), 2.41 (s, 3H), 2.35 (s, 3H); ^13^C-NMR: 159.9, 157.5, 152.8, 143.0, 139.4, 111.9, 108.0, 107.7, 98.2, 55.0, 46.2, 45.3, 24.8. HR-MS (ESI). Calcd for C_15_H_20_N_3_O (M^+^ + 1): *m/z* 258.1606. Found: *m/z* 258.1618. A hydrobromide salt: m.p. 148–150 °C. Anal. Calcd for C_15_H_19_N_3_O•HBr•H_2_O: C, 50.57; H, 6.22; N, 11.79. Found: C, 50.41; H, 6.42; N, 11.39.

*2-Methyl-6-(4-methylpiperazin-1-yl)-4-(2-thienyl)pyridine* (**56**). Yield 6% (free base); an oil; ^1^H-NMR: δ 7.41 (d, *J* = 3.2 Hz, 1H), 7.34 (d, *J* = 4.8 Hz, 1H), 7.09 (m, 1H), 6.74 (s, 1H), 6.63 (s, 1H), 3.61 (t, *J* = 4.8 Hz, 4H), 2.55 (t, *J* = 4.8 Hz, 4H), 2.43 (s, 3H), 2.36 (s, 3H). A hydrobromide salt: m.p. 132–134 °C. Anal. Calcd for C_15_H_19_N_3_S•HBr: C, 50.85; H, 5.68; N, 11.86. Found: C, 51.10; H, 5.69; N, 11.53.

*6-(3-Furyl)-2-(4-methylpiperazin-1-yl)pyridine* (**57**). Yield 80% (free base); an oil; ^1^H-NMR: δ 7.99 (s, 1H), 7.49 (m, 1H), 7.30 (m, 2H), 6.85 (m, 1H), 6.54 (m, 1H), 3.64 (t, *J* = 5.0 Hz, 4H), 2.57 (t, *J* = 5.0 Hz, 4H), 2.37 (s, 3H); ^13^C-NMR: δ 159.1, 149.8, 143.4, 141.1, 138.0, 127.7, 109.5, 108.8, 105.0, 54.9, 46.3, 45.5. HR-MS (ESI). Calcd for C_14_H_18_N_3_O (M^+^ + 1): *m/z* 244.1461. Found: *m/z* 244.1450. A hydrobromide salt: m.p. >250 °C. Anal. Calcd for C_14_H_17_N_3_O•2HBr•2H_2_O: C, 38.12; H, 5.25; N, 9.52. Found: C, 38.23; H, 5.33; N, 9.72.

*2-(4-Methylpiperazin-1-yl)-6-(3-thienyl)pyridine* (**58**). Yield 42% (free base); an oil; ^1^H-NMR: δ 7.86 (m, 1H), 7.63 (m, 1H), 7.49 (t, *J* = 4.0 Hz, 1H), 7.33 (m, 1H), 6.97 (d, *J* = 4.0 Hz, 1H), 6.54 (d, *J* = 4.0 Hz, 1H), 3.64 (t, *J* = 5.0 Hz, 4H), 2.53 (t, *J* = 5.0 Hz, 4H), 2.35 (s, 3H); ^13^C-NMR: δ 159.0, 154.4, 143.0, 138.2, 126.4, 125.7, 123.0, 109.7, 105.2, 55.0, 46.3, 45.1. A hydrobromide salt: m. p. >250 °C. Anal. Calcd for C_14_H_17_N_3_S•2HBr•H_2_O: C, 39.09; H, 4.69; N, 9.77. Found: C, 39.11; H, 4.79; N, 9.90.

*2-(2-Furyl)-6-(4-methylpiperazin-1-yl)pyridine* (**59**). Yield 38% (free base); an oil; ^1^H-NMR: δ 8.17 (d, *J* = 5.2 Hz, 1H), 7.48 (d, *J* = 1.6 Hz, 1H), 6.92 (m, 1H), 6.85 (d, *J* = 5.2 Hz, 1H), 6.77 (d, *J* = 3.6 Hz, 1H), 6.57 (d, *J* = 3.6 Hz, 1H), 3.61 (t, *J* = 5.2 Hz, 4H), 2.52 (t, *J* = 5.2 Hz, 4H), 2.34 )s, 3H); ^13^C-NMR: δ 160.1, 152.3, 148.3, 143.1, 138.8, 111.9, 108.4, 107.9, 100.9, 54.9, 46.2, 45.2. A hydrobromide salt: m.p. >250 °C. Anal. Calcd for C_14_H_17_N_3_O•2HBr•2H_2_O: C, 38.12; H, 5.25; N, 9.52. Found: C, 37.94; H, 5.02; N, 9.54.

### 4.11. Synthesis of Substituted Benzenes ***60**–**71***

A mixture of 1,3-dibromobenzene, 1,3-dibromo-5-methylbenzene or 1,3-dibromo-5-*n*-butylbenzene (21 mmol) and *N*-methylpiperazine (0.8 mL), 7.0 mmol) in toluene (20 mL) was stirred under a nitrogen atmosphere and treated quickly with 2,2′-bis(diphenylphosphino)-1,1′-binaphthyl (BINAP, 0.13 g, 0.21 mmol) and tris (dibenzylideneacetone)dipalladium(0) (Pd_2_dba_3_, 0.05 g, 0.06 mmol). The flask was refilled with nitrogen, capped with a rubber septum, and 1,8-diazabicyclo[5.4.0]undec-7-ene (DBU, 2.6 mL, 17.5 mmol) was added via a syringe. The mixture was warmed to 60 °C before treatment with sodium *tert*-butoxide (0.01 g, 0.05 mmol) in one portion. The mixture was heated at 110 °C for 12 h, then cooled and partitioned between ethyl acetate (EtOAc, 40 mL) and water (40 mL). The aqueous layer was extracted with EtOAc (40 mL). The organic layers were combined and washed with hydrochloric acid (1.6 M, 2 × 25 mL). The aqueous acidic layer containing product was made basic with NaOH solution (1M, 50 mL) and the mixture was extracted with EtOAc (2 × 40 mL). The extract was washed with brine (25 mL), dried over magnesium sulfate, filtered and concentrated under reduced pressure to provide an analytically pure product.

*1-(3-Bromophenyl)-4-methylpiperazine*; yield 30%; an oil; ^13^C-NMR: δ 152.6, 130.5, 123.4, 122.3, 118.8, 114.5, 55.1, 48.8.46.3. HR-MS (ESI). Calcd for C_11_H_15_BrN_2_ (M^+^ + 1): *m/z* 255.0497. Found: *m/z* 255.0493.

*1-(3-Bromo-5-methylphenyl)-4-methylpiperazine*; yield 54%; an oil; ^13^C-NMR: δ 152.5, 140.6, 131.1, 123.2, 116.1, 115.4, 55.2, 48.9, 46.3, 21.7. HR-MS (ESI). Calcd for C_12_H_17_BrN_2_ (M^+^ + 1): *m/z* 269.0653. Found: 269.0653.

*1-(3-Bromo-5-n-butylphenyl)-4-methylpiperazine*; yield 32%; an oil; ^13^C-NMR: δ 152.3, 145.5, 122.9, 122.4, 116.0, 114.7, 55.0, 48.7, 46.1, 35.8, 33.4, 22.3, 13.9. HR-MS (ESI). Calcd for C_15_H_24_BrN_2_ (M^+^ + 1): *m/z* 311.1123. Found: *m/z* 311.1133.

A bromophenylpiperazine derivative (1.2 mmol), obtained as described above, was allowed to react with furan-3-boronic acid, furan-2-boronic acid, thiophene-3-boronic acid or thiophene-2-boronic acid (1.8 mmol) in DMF (5 mL) and water (0.5 mL) in the presence of tetrakis(triphenylphosphine)palladium(0) (0.09 mmol) and potassium carbonate (0.5 g, 3.6 mmol) at 90 °C under a nitrogen atmosphere in a sealed tube for 12 h. After cooling, the mixture was filtered through Celite and the adsorbent was washed with EtOAc (20 mL). Concentration followed by chromatography eluting with hexanes/ether (4:1) gave the oily product **60**–**71** that was additionally purified by crystallization of a hydrobromide salt from ethanol.

*1-[3-(3-Furyl)phenyl]-4-methylpiperazine* (**60**). Yield 34%; ^1^H-NMR: δ 7.70 (s, 1H), 7.46 (s, 1H), 7.26 (t, *J* = 5.6 Hz, 1H), 7.03 (s, 1H), 6.99 (d, *J* = 7.6 Hz, 1H), 6.85 (m, 1H), 6.68 (s, 1H), 3.27 (t, *J* = 4.8 Hz, 4H), 2.62 (t, *J* = 4.8 Hz, 4H), 2.38 (s, 3H); ^13^C-NMR: δ 151.9, 143.6, 138.7, 133.5, 129.7, 127.1, 117.8, 115.0, 113.9, 109.2, 55.3, 49.3, 46.3. HR-MS (ESI). Calcd for C_15_H_19_N_2_O (M^+^ + 1): *m/z* 243.1497. Found: *m/z* 243.1492. A hydrobromide salt: m.p. 215–217 °C. Anal. Calcd for C_15_H_18_N_2_O•2HBr: C, 44.58; H, 4.99; N, 6.93. Found: C, 44.31; H, 4.99; N, 6.62.

*1-[3-(3-Furyl)-5-methylphenyl]-4-methylpiperazine* (**61**). Yield 25%; ^1^H-NMR: δ 7.68 (s, 1H), 7.44 (s, 1H), 6.85 (s, 1H), 6.82 (s, 1H), 6.67 (s, 2H), 3.23 (t, *J* = 4.8 Hz, 4H), 2.59 (t, *J* = 4.8 Hz, 4H), 2.34 (2s, 6H); ^13^C-NMR: δ 151.7, 143.4, 139.2, 138.5, 133.1, 126.9, 118.6, 115.7, 111.1, 109.1, 55.1, 49.2, 46.1, 21.7. HR-MS (ESI). Calcd for C_16_H_21_N_2_O (M^+^ + 1): *m/z* 257.1654. Found: *m/z* 257.1657. A hydrobromide salt: m.p. 230–232 °C. Anal. Calcd for C_16_H_20_N_2_O•2HBr: C, 45.96; H, 5.30; N, 6.70. Found: C, 46.30; H, 5.28; N, 6.34.

*1-[3-n-Butyl-5-(3-furyl)phenyl]-4-methylpiperazine* (**62**). Yield 70%; ^1^H-NMR: δ 7.69 (s, 1H), 7.43 (t, *J* = 1.6 Hz, 1H), 6.85 (s, 1H), 6.82 (s, 1H), 6.67 (m, 2H), 3.23 (t, *J* = 5.2 Hz, 4H), 2.57 (m, 6H), 1.61 (m, 2H), 1.37 (m, 2H), 0.93 (t, *J* = 7.2 Hz, 3H); ^13^C-NMR: δ 151.8, 144.4, 143.4, 138.5, 133.1, 127.1, 118.1, 115.4, 111.3, 109.2, 55.3, 49.4, 46.2, 36.2, 33.8, 22.6, 14.1. HR-MS (ESI). Calcd for C_19_H_27_N_2_O (M^+^ + 1): *m/z* 299.2123. Found: *m/z* 299.2136. A hydrobromide salt: m.p. 215–219 °C. Anal. Calcd for C_19_H_26_N_2_O•2HBr•0.5H_2_O: C, 48.63; H, 6.23; N, 5.97. Found: C, 48.55; H, 6.14; N, 6.20.

*1-[3-(2-Furyl)phenyl]-4-methylpiperazine* (**63**). Yield 24%; ^1^H-NMR: δ 7.44 (s, 1H), 7.27 (m, 2H), 7.16 (d, *J* = 7.6 Hz, 1H), 6.84 (d, *J* = 7.6 Hz, 1H), 6.62 (m, 1H), 6.45 (m, 1H), 3.27 (t, *J* = 4.6 Hz, 4H), 2.60 (t, *J* = 4.6 Hz, 4H), 2.36 (s, 3H); ^13^C-NMR: δ 154.5, 151.7, 142.0, 131.8, 129.6, 115.5, 115.4, 111.7, 111.5, 105.1, 55.3, 49.2, 46.3. HR-MS (ESI): Calcd for C_15_H_19_N_2_O (M^+^ + 1): *m/z* 243.1497. Found: *m/z* 243.1487. A hydrobromide salt: m.p. 240–242 °C. Anal. Calcd for C_15_H_18_N_2_O•2HBr•0.5H_2_O: C, 43.61; H, 5.12; N, 6.78. Found: C, 43.24; H, 5.12; N, 7.15.

*1-[3-(2-Furyl)-5-methylphenyl]-4-methylpiperazine* (**64**). Yield 15%; ^1^H-NMR: δ 7.46 (s, 1H), 7.09 (s, 1H), 7.03 (s, 1H), 6.69 (m, 1H), 6.63 (m, 1H), 6.47 (m, 1H), 3.31 (t, *J* = 5.0 Hz, 4H), 2.67 (t, *J* = 5.0 Hz, 4H), 2.42 (s, 3H), 2.36 (s, 3H); ^13^C-NMR: δ 154.4, 151.4, 141.7, 139.1, 131.5, 116.6, 116.2, 111.5, 108.9, 104.8, 54.9, 48.9, 45.8, 21.8. HR-MS (ESI). Calcd for C_16_H_21_N_2_O (M^+^ + 1): *m/z* 257.1654. Found: *m/z* 257.1651. A hydrobromide salt: m.p. 245–249 °C. Anal. Calcd for C_16_H_20_N_2_O•2HBr: C, 45.97; H, 5.30; N, 6.70. Found: C, 45.64; H, 5.26; N, 6.81.

*1-[3-n-Butyl-5-(2-furyl)phenyl]-4-methylpiperazine* (**65**). Yield 76%; ^1^H-NMR: δ 7.44 (s, 1H), 7.07 (s, 1H), 7.01 (s, 1H), 6.68 (m, 1H), 6.61 (d, *J* = 3.2 Hz, 1H), 6.45 q, *J* = 3.2 Hz, 1H), 3.26 (t, *J* = 4.8 Hz, 4H), 2.59 (t, *J* = 5.6 Hz, 6H), 2.36 (s, 3H), 1.62 (m, 2H), 1.37 (m, 2H), 0.93 (t, *J* = 7.2 Hz, 3H); ^13^C-NMR: δ 154.7, 151.7, 144.4, 141.9, 131.7, 116.1, 116.0, 111.7, 109.2, 105.0, 55.3, 49.3, 46.2, 36.3, 33.8, 22.6, 14.2. HR-MS (ESI). Calcd for C_19_H_27_N_2_O (M^+^ + 1): *m/z* 299.2123. Found: *m/z* 299.2124. A hydrobromide salt: m.p. 180–182 °C. Anal. Calcd for C_19_H_26_N_2_O•HBr: C, 60.16; H, 7.17; N, 7.38. Found: C, 60.37; H, 7.15; N, 7.54.

*1-Methyl-4-[3-(3-thienyl)phenyl]piperazine* (**66**). Yield 25%; ^1^H-NMR: δ 7.42 (t, *J* = 2.0 Hz, 1H), 7.36 (d, *J* = 2.0 Hz, 2H), 7.29 (t, *J* = 8 Hz, 1H), 7.14 (s, 1H), 7.09 (d, *J* = 8.0 Hz, 1H), 6.87 (m, 1H), 3.27 (t, *J* = 4.8 Hz, 4H), 2.62 (t, *J* = 4.8 Hz, 4H), 2.38 (s, 3H); ^13^C-NMR: δ 151.8, 143.1, 137.0, 129.7, 126.7, 126.2, 120.5, 118.5, 115.2, 114.6, 55.3, 49.3, 46.3. HR-MS (ESI). Calcd for C_15_H_19_N_2_S (M^+^ + 1): *m/z* 259.1269. Found: *m/z* 259.1269. A hydrobromide salt: m.p. 200–205 °C. Anal. Calcd for C_15_H_18_N_2_S•3HBr: C, 35.95; H, 4.22; N, 5.66. Found: C, 35.97; H, 4.19; N, 5.66.

*1-Methyl-4-[3-methyl-5-(3-thienyl)phenyl]piperazine* (**67**). Yield 25%; ^1^H-NMR: δ 7.40 (s, 1H), 7.35 (s, 2H), 6.95 (s, 1H), 6.92 (s, 1H), 6.70 (s, 1H), 3.25 (t, *J* = 4.8 Hz, 4H), 2.61 (t, *J* = 4.8 Hz, 4H), 2.35 (2s, 6H); ^13^C-NMR: δ 151.9, 143.1, 139.3, 136.9, 126.8, 126.0, 120.3, 119.4, 116.0, 111.9, 55.3, 49.3, 46.2, 22.0. HR-MS (ESI). Calcd for C_16_H_21_N_2_S (M^+^ + 1): *m/z* 273.1425. Found: *m/z* 273.1432. A hydrobromide salt: m.p. 245–247 °C. Anal. Calcd for C_16_H_20_N_2_S•1.5HBr•2H_2_O: C, 44.89; H, 4.85; N, 6.03. Found: C, 44.71; H, 5.23; N, 6.42.

*1-[3-n-Butyl-5-(3-thienyl)phenyl]-4-methylpiperazine* (**68**). Yield 23%; ^1^H-NMR: δ 7.40 (t, *J* = 2.4 Hz, 1H), 7.35 (m, 2H), 6.96 (s, 1H), 6.92 (s, 1H), 6.71 (s, 1H), 3.26 (t, *J* = 4.8 Hz, 4H), 2.60 (m, 6H), 2.36 (s, 3H), 1.62 (m, 2H), 1.39 (m, 2H), 0.93 (t, *J* = 7.6 Hz, 3H); ^13^C-NMR: δ 151.9, 144.5, 143.3, 136.8, 126.9, 126.0, 120.3, 118.9, 115.6, 112.1, 55.4, 49.5, 46.3, 36.3, 33.9, 22.7, 14.2. HR-MS (ESI). Calcd for C_19_H_27_N_2_S (M^+^ + 1): *m/z* 315.1895. Found: *m/z* 315.1910. A hydrobromide salt: m.p. 215–217 °C. Anal. Calcd for C_19_H_26_N_2_S•2HBr: C, 47.91; H, 5.93; N, 5.93. Found: C, 47.60; H, 6.19; N, 6.20.

*1-Methyl-4-[3-(2-thienyl)phenyl]piperazine* (**69**). Yield 23%; ^1^H-NMR: δ 7.27 (m, 3H), 7.15 (s, 1H), 7.10 (d, *J* = 8.4 Hz, 1H), 7.06 (m, 1H), 6.86 (m, 1H), 3.26 (t, *J* = 4.8 Hz, 4H), 2.59 (t, *J* = 4.8 Hz, 4H), 2.36 (s, 3H); ^13^C-NMR: δ 151.7, 144.9, 135.2, 129.6, 127.8, 124.6, 123.0, 117.7, 115.2, 113.8, 55.1, 49.0, 46.1. HR-MS (ESI). Calcd for C_15_H_19_N_2_S (M^+^ + 1): *m/z* 259.1269. Found: *m/z* 259.1279. A hydrobromide salt: m.p. >250 °C. Anal. Calcd for C_15_H_18_N_2_S•2HBr: C, 42.87; H, 4.80; N, 6.67. Found: C, 42.55; H, 4.78; N, 6.65.

*1-Methyl-4-[3-methyl-5-(2-thienyl)phenyl]piperazine* (**70**). Yield 27%; ^1^H-NMR: δ 7.26 (m, 2H), 7.05 (t, *J* = 3.6 Hz, 1H), 6.97 (m, 2H), 6.69 (s, 1H), 3.26 (t, *J* = 4.8 Hz, 4H), 2.62 (t, *J* = 4.8 Hz, 4H), 2.34 (s, 3H), 2.36 (s, 3H); ^13^C-NMR: δ 151.7, 145.0, 139.3, 135.1, 127.8, 124.4, 123.0, 118.7, 116.2, 111.1, 55.1, 49.1, 46.1, 21.7. HR-MS (ESI). Calcd for C_16_H_21_N_2_S (M^+^ + 1): *m/z* 273.1425. Found: *m/z* 273.1434. A hydrobromide salt: m.p. >250 °C. Anal. Calcd for C_16_H_20_N_2_S•2HBr•0.5H_2_O: C, 43.36; H, 5.23; N, 6.23. Found: C, 43.47; H, 5.15; N, 6.61.

*1-[3-n-Butyl-5-(2-thienyl)phenyl]-4-methylpiperazine* (**71**). Yield 26%; ^1^H-NMR: δ 7.25 (m, 2H), 7.05 (m, 1H), 6.97 (t, *J* = 2.0 Hz, 1H), 6.94, (s, 1H), 6.70 (s, 1H), 3.25 (t, *J* = 5.2 Hz, 4H), 2.59 (m, 6H), 2.60 (s, 3H), 1.59 9M, 2H), 1.37 (m, 2H), 0.93 (t, *J* = 7.2 Hz, 3H); ^13^C-NMR: δ 151.9, 145.4, 144.6, 135.2,127.9, 124.6, 123.1, 118.3, 115.9, 111.5, 55.3, 49.4, 46.3, 36.2, 33.8, 22.7, 14.2. HR-MS (ESI). Calcd for C_19_H_27_N_2_O (M^+^ + 1): *m/z* 315.1895. Found: *m/z* 315.1885. A hydrobromide salt: m.p. 234–236 °C. Anal. Calcd for C_19_H_26_N_2_S•1.5HBr: C, 52.36; H, 6.36; N, 6.34. Found: C, 52.01; H, 6.40; N, 6.50.

## 5. Conclusions

Piperazin-1-yl substituted unfused heterobiaryls incorporating 4-(3-furyl) moiety demonstrate high affinities for 5-HT_7_ receptor. The SAR studies on the model ligand 4-(3-furyl)-2-(4-methylpiperazin-1-yl)pyrimidine (**2**) (*K*_i_ = 7.2 nM) revealed that introduction of an alkyl group to position 6 of the pyrimidine leads to increase in the binding affinity, however, a substituent at position 5 is highly detrimental. It was also shown that the pyrimidine core can be replaced with a pyridine ring without a significant loss of the binding affinity, although piperazine moiety is optimal, as its replacement results in decreased 5-HT_7_ affinity. The selected 6-ethylpyrimidine **12** (*K*_i_ = 7.1 nM) and 6-butylpyrimidine **13** (*K*_i_ = 1.6 nM) ligands demonstrated 5-HT_7_ antagonistic properties in cAMP functional test (*K*_B_ = 12 and 60 nM, respectively). The extended selectivity studies with use of other types of serotonin (5-HT_1A_, 5-HT_2A_, 5-HT_6_) and dopamine D_2_ receptors showed that compound **12** displays also pronounced affinity for 5-HT_2A_ receptors (*K*_i_ = 12 nM), which is consistent with our previous studies of piperazin-1-yl substituted heterobiaryl derivatives. 6-Butylpyrimidine analog **13** lacks selectivity towards 5-HT_2A_, 5-HT_6_ and dopamine D_2_ receptors. In summary, piperazin-1-yl substituted heterobiaryls constitute a potent group of serotonergic agents that could be further studied in animal models of CNS disorders.

## Figures and Tables

**Table 1:** 5-HT_7_ binding affinities of pyrimidine derivatives **1**–**41**.

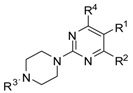	R^1^	R^2^	R^3^	R^4^	*K*_i_ ± S.D. (nM)
**1**	H	3-furyl	H	H	9 ± 2
**2** ^a^	H	3-furyl	Me	H	7.2 ± 1.4
**3**	H	3-furyl	Et	H	10 ± 1
**4**	H	3-furyl	Pr	H	5.3 ± 0.9
**5**	H	3-furyl	Bn	H	25 ± 3
**6**	H	3-furyl	Bz	H	1405 ± 173
**7**	H	3-furyl	(CH_2_)_3_Ph	H	1.6 ± 0.2
**8**	H	3-furyl	(CH_2_)_2_OH	H	8.5 ± 1.1
**9**	H	3-furyl	(CH_2_)_2_COOEt	H	8.9 ± 2.1
**10**	H	3-furyl	(CH_2_)_5_COOEt	H	1.4 ± 0.3
**11** ^a^	H	3-furyl	Me	Me	3.9 ± 0.7
**12**	H	3-furyl	Me	Et	7.1 ± 1.4
**13**	H	3-furyl	Me	*^n^*Bu	1.6 ± 0.2
**14**	H	3-furyl	Me	*^n^*Hexyl	3.3 ± 0.5
**15**	H	3-furyl	Me	*^s^*Bu	2.7± 0.5
**16**	H	3-furyl	Me	CF_3_	8.3 ± 1.7
**17**	H	3-furyl	Me	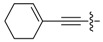	18 ± 3
**18** ^a^	H	3-furyl	Me	3-furyl	22 ± 2
**19**	H	3-furyl	Me	N(Me)_2_	>10,000
**20**	H	3-furyl	Me	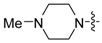	>10,000
**21**	*^n^*Pr	3-furyl	Me	H	1334 ± 232
**22**	*^n^*Pr	3-furyl	Me	*^n^*Bu	1250 ± 116
**23**	H	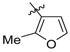	Me	H	1920 ± 373
**24**	H	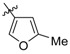	Me	H	462 ± 54
**25**	H	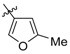	Me	*^n^*hexyl	12 ± 2
**26** ^b^	H	2-furyl	Me	H	1021 ± 96
**27**	H	2-furyl	Me	Et	380 ± 44
**28**	H	2-furyl	Me	*^n^*Bu	450 ± 63
**29**	H	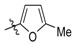	Me	H	4230 ± 680
**30**	H	3-thienyl	Me	H	83 ± 9
**31** ^c^	H	3-thienyl	Me	*^n^*Bu	15 ± 3
**32** ^a,b^	H	2-thienyl	Me	H	480 ± 65
**33** ^d^	H	2-OH-C_6_H_4_	Me	H	>10,000
**34** ^d^	H	3-OH-C_6_H_4_	Me	H	>10,000
**35** ^d^	H	4-OH-C_6_H_4_	Me	H	>10,000
**36**	H	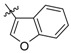	Me	H	7460 ± 1230
**37**	H	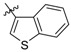	Me	H	5280 ± 820
**38**	H	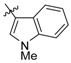	Me	H	1530 ± 125
**39** ^d^	H	3-pyridinyl	Me	H	780 ± 94
**40**	H	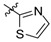	Me	H	>10,000
**41**	H	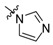	Me	H	1980 ± 275

^a^: Ref. [24]; ^b^: Ref. [29]; ^c^: Ref. [30]; ^d^: Ref. [27].

**Table 2:** Affinities of pyrimidines **42**–**47** for 5-HT_7_ receptor.

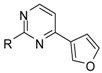	R	*K*_i_ ± S.D. (nM)
**42**	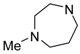	148 ± 17
**43**	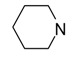	>10,000
**44**	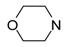	>10,000
**45**	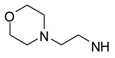	2460 ± 343
**46**	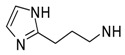	1280 ± 187
**47**	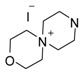	299 ± 42

**Table 3:** Affinities of substituted quinazolines **48**–**50** for 5-HT_7_ receptor.

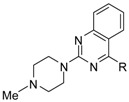	R	*K*_i_ ± SD (nM)
**48**	3-furyl	1910 ± 235
**49**	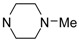	7540 ± 1140
**50**	(CH_2_)_2_N(Me)_2_	>10,000

**Table 4:** Binding affinities of substituted pyridines **51**–**59** for 5-HT_7_ receptor (see Scheme 6, Scheme 7 and Scheme 8).

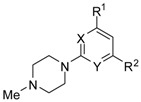	R^1^	R^2^	X	Y	*K*_i_ ± S.D. (nM)
**51**	H	3-furyl	N	CH	17 ± 2
**52**	H	2-furyl	N	CH	307 ± 42
**53**	H	2-thienyl	N	CH	396 ± 55
**54**	Me	3-furyl	N	CH	8 ± 1
**55**	Me	2-furyl	N	CH	139 ± 22
**56**	Me	2-thienyl	N	CH	117 ± 12
**57**	H	3-furyl	CH	N	11 ± 2
**58**	H	3-thienyl	CH	N	79 ± 14
**59**	H	2-furyl	CH	N	1135 ± 154

**Table 5:** Binding affinities of substituted benzenes **60**–**71** for 5-HT_7_ receptor.

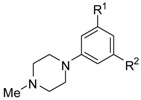	R^1^	R^2^	K_i_ ± S.D. (nM)
**60**	H	3-furyl	31 ± 4
**61**	Me	3-furyl	37 ± 3
**62**	*^n^*Bu	3-furyl	106 ± 9
**63**	H	2-furyl	914 ± 121
**64**	Me	2-furyl	312 ± 25
**65**	*^n^*Bu	2-furyl	743 ± 66
**66**	H	3-thienyl	342 ± 51
**67**	Me	3-thienyl	47 ± 6
**68**	*^n^*Bu	3-thienyl	263 ± 19
**69**	H	2-thienyl	542 ± 66
**70**	Me	2-thienyl	297 ± 42
**71**	*^n^*Bu	2-thienyl	734 ± 59

**Table 6:** Extended results for compounds **12** and **13**.

	*K*_i_ ± S.D. (nM)				
	D_2_	5-HT_1A_	5-HT_2A_	5-HT_6_	5-HT_7_
**12**	466 ± 39	1501 ± 164	12 ± 2	121 ± 14	7.1 ± 1.4
**13**	37 ± 4	637 ± 53	10 ± 1	12 ± 2	1.6 ± 0.2
